# Ultrafast, autonomous self-healable iontronic skin exhibiting piezo-ionic dynamics

**DOI:** 10.1038/s41467-022-35434-8

**Published:** 2022-12-13

**Authors:** Elvis K. Boahen, Baohai Pan, Hyukmin Kweon, Joo Sung Kim, Hanbin Choi, Zhengyang Kong, Dong Jun Kim, Jin Zhu, Wu Bin Ying, Kyung Jin Lee, Do Hwan Kim

**Affiliations:** 1 Department of Chemical Engineering, Hanyang University, Seoul, 04763 Republic of Korea; 2 Department of Chemical Engineering and Applied Chemistry, Chungnam National University, Daejeon, 34134 Republic of Korea; 3 Ningbo Institute of Materials Technology and Engineering, Chinese Academy of Sciences, Ningbo, 315201 People’s Republic of China; 4 Institute of Nano Science and Technology, Hanyang University, Seoul, 04763 Republic of Korea

**Keywords:** Polymers, Polymers, Organic molecules in materials science

## Abstract

The self-healing properties and ionic sensing capabilities of the human skin offer inspiring groundwork for the designs of stretchable iontronic skins. However, from electronic to ionic mechanosensitive skins, simultaneously achieving autonomously superior self-healing properties, superior elasticity, and effective control of ion dynamics in a homogeneous system is rarely feasible. Here, we report a Cl-functionalized iontronic pressure sensitive material (CLiPS), designed via the introduction of Cl-functionalized groups into a polyurethane matrix, which realizes an ultrafast, autonomous self-healing speed (4.3 µm/min), high self-healing efficiency (91% within 60 min), and mechanosensitive piezo-ionic dynamics. This strategy promotes both an excellent elastic recovery (100%) and effective control of ion dynamics because the Cl groups trap the ions in the system via ion-dipole interactions, resulting in excellent pressure sensitivity (7.36 kPa^−1^) for tactile sensors. The skin-like sensor responds to pressure variations, demonstrating its potential for touch modulation in future wearable electronics and human–machine interfaces.

## Abstract (web summary)

Sensing properties of the human skin are inspiring for the design of stretchable iontronics but achieving self-healing properties, elasticity and effective control of ion dynamics at the same time is challenging. Here, the Authors report a Cl-functionalized iontronic pressure sensitive material and realize good self-healing properties and mechanosensitive piezo-ionic dynamics.

## Introduction

The human skin is a crucial body organ that possesses a self-healing capability to restore its functionalities upon external injury, as well as a mechanosensory system for the detection of pressure, strain, and torsion based on iontronics^1–4^. Inspired by the sensory structure of the human skin, several research groups have reported electronic skins based on iontronic materials owing to their high noise immunity, exceptional spatial resolution, and excellent response to both static and dynamic stimuli^5–7^. However, such iontronic materials are vulnerable to unexpected mechanical damage caused by continual wear and tear, leading to broken functionalities and limited device longevity. Thus, similar to the human skin, self-healing is an important intrinsic property essential for restoring broken functionalities to ensure stability and increase the operational lifespan of a device. However, previously reported self-healing iontronic polymers^8–10^ usually present a compromise between the autonomous self-healing speed and elasticity.

Conventionally, elastomers comprise strong covalently bonded polymer chains to achieve superior elasticity, which enables the elastomers to restore to their original states after deformation owing to the entropic force^8^. In contrast, autonomous self-healing mechanisms often rely on the rearrangement and reorganization of intrinsic weak dynamic bonds at the damage site^11^. Polymer chains with weak dynamic bonds designed for fast room-temperature self-healing invariably impart poor elasticity. Furthermore, the iontronic sensing mechanism most often generates a high initial capacitance due to the limited control of mobile ion dynamics, which results in poor sensitivity^3,6^. To enhance the control of ion dynamics, previous studies have employed inorganic–based heterogeneous systems to realize ion confinement effect. For instance, our group^4^ and Park et al.^12^ recently introduced silica nanoparticles and Ti_3_C_2_T_x_ MXene, respectively, to achieve ion confinement via hydrogen bonding, leading to tremendously enhanced device sensitivity. However, the design of such heterogeneous systems can hardly achieve autonomously superior self-healing properties owing to the restricted mobility of polymer chains caused by the slow-moving nanomaterials attached to the polymer, thereby creating a significantly sluggish self-healing speed and decreased self-healing efficiency^13,14^. Therefore, designing iontronic systems with simultaneous superior self-healing properties, good elasticity, and effective ion dynamics, is challenging.

Here, we present a Cl-functionalized (CL) iontronic pressure-sensitive material (CLiPS) via the introduction of Cl-functionalized groups (Cl groups) into a polyurethane (PU) matrix, which exhibits not only excellent autonomous self-healing properties but also mechanosensitive piezo-ionic dynamics. The design concept involves strategic selection of high chain mobility isophorone diisocyanate (IPDI) and dynamic disulfide bonds^15^ to construct the backbone of the PU structure, which are key factors in achieving autonomous self-healing properties at room temperature. In addition, excellent elastic recovery can be achieved owing to the intrinsic toughness of the Cl groups. Moreover, with the inclusion of the (1-ethyl-3-methylimidazolium bis(trifluoromethylsulfonyl)imide ([EMIM]^+^[TFSI]^–^)) ionic liquid (IL), a piezo-ionic mechanism (demonstrating mechanosensitive ion trap and release behavior) is established owing to the ion–dipole interactions between the Cl groups and the ion pairs. The CLiPS-based electronic skin (e-skin) designed in this study provides the framework for the simultaneous realization of fast self-healing properties and the mechanosensitive ion dynamics in a homogeneous system.

## Results

**Fig. 1:**
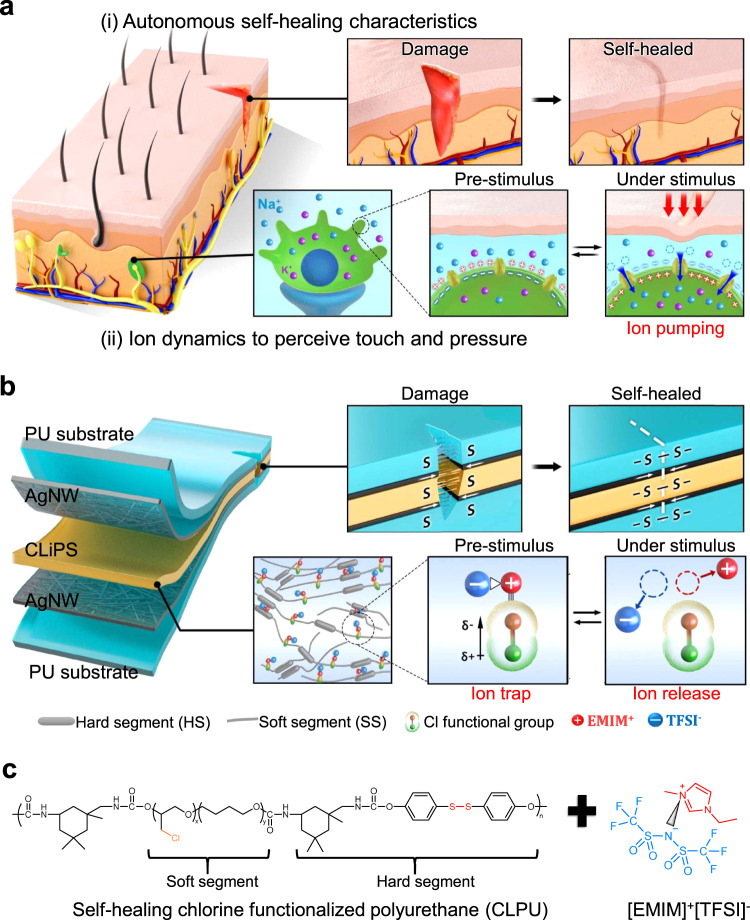
Molecular structure of the skin and conceptual design mechanism of CLiPS. **a** Schematics of the self-healing properties and biological mechanoreceptor of the human skin. The human skin achieves strong autonomous self-healing upon damage owing to the activities of blood cells and platelets. It also perceives external stimuli via the generation of an action potential resulting from ion dynamics. **b** CLiPS-based device architecture illustrating autonomous self-healing and tractive self-healing of electrodes, as well as the molecular structure emulating the ion dynamics of the human skin based on the trap and release mechanism. **c** Chemical structure representation of the design rule of CLiPS.

As illustrated in Fig. 1a, biological skin is capable of autonomously self-healing damaged areas owing to the activities of blood cells and platelets^16,17^. This function ensures rejuvenation and maintenance of the skin to retain its functionality for a prolonged period. Furthermore, the unique functional traits of representative mechanoreceptors in the human skin exhibit ultrasensitive pressure-sensing efficiencies over a wide range of pressures. At pre-stimulus conditions, these cells exhibit a polarized resting membrane potential, which is sustained across the cell membrane owing to the concentration gradient between Na^+^ and K^+^ ions, along with closed ion channels. Under a stimulus, open ion channels induce the pumping of Na^+^ ions, leading to the depolarization of the cell membrane and generation of an action potential which is transmitted to the central nervous system^18–20^.

Inspired by the self-healing properties and ion dynamics of the mechanoreceptors in human skin, Fig. 1b presents the conceptual design of the CLiPS that emulates the remarkable functionalities of human skin described above. As depicted in Fig. 1c, the CLiPS is designed using Cl-functionalized polyurethane (CLPU) matrix consisting of high chain mobility IPDI and dynamic disulfide bonds that guarantee autonomous self-healing without external stimuli^15^. In addition, tractive self-healing of the electrodes were achieved at room temperature owing to the excellent self-healing properties of CLPU, resulting in the autonomous self-healing of the entire device structure. With the incorporation of ILs, the self-healing speed can be enhanced tremendously owing to the plasticization of the ions on the CLPU chains. Moreover, the [EMIM]^+^[TFSI]^–^ ion pairs are trapped to the Cl groups via ion–dipole interactions, initiating the trap and release phenomena. Notably, Cl groups comprise of highly electronegative Cl atoms that can generate a high dipole moment during C–Cl bond formation^21,22^. The dipole phenomenon arises as the more electronegative Cl atom pulls the bonded shared pair of electrons toward itself, making it partially negatively charged and the carbon atom partially positively charged. Therefore, the partially negative Cl atom interacts with the [EMIM]^+^ cation through ion–dipole interactions, resulting in the trapping of ion pairs. Thus, for better ion trapping effects, it is necessary to increase the number of Cl groups; however, the numerous Cl groups may drastically reduce the self-healing speed.

### Rational design and molecular optimization of ultrafast, autonomous self-healing CLiPS

To establish the optimum conditions for efficient ion trapping and a fast self-healing speed in the CLiPS, various CLPUs were synthesized (Supplementary Note 1 and Supplementary Fig. 1) with a stepwise increase in the content of Cl groups in the soft segment of the PU matrix (molecular ratios of the soft segment of each sample are listed in Supplementary Table 1), as described in the experimental section, while analyzing the self-healing speed of each sample, (the samples were labeled with respect to the Cl groups composition; E3 had the lowest composition, and E7 had the highest composition). As postulated, there was a drastic reduction in the self-healing speed of the CLPUs as the content of Cl groups increased, with E3 and E7 exhibiting the highest and lowest self-healing speeds, respectively. This tendency can be attributed to the intrinsic toughness properties of the Cl groups, which decrease the polymer chain mobility, causing a reduction in the self-healing speed as the Cl groups proportion increase. The mechanical properties of the CLPUs (Supplementary Fig. 2, Supplementary Table 2, and Supplementary Table 3) reflected the intrinsic toughness of the Cl groups, where the elastic modulus increased as the Cl groups content increased. Moreover, as depicted in Supplementary Fig. 3a, the glass transition temperatures (T_g_) of the CLPUs increased as the Cl groups content increased. These results strongly support the claim that Cl groups impart intrinsic toughness properties that can influence the polymer chain mobility of the CLPUs.

Even with the addition of a 30 wt% IL to the CLPU, the mechanical properties demonstrated relative reduction while maintaining the trend with respect to the Cl groups content. The relative decrease in the elastic modulus is attributed to the plasticization effect of ions, which causes facile movements of polymer chains, thereby yielding a lower modulus and higher elongation at break^3^. The plasticization effect was also confirmed based on the decrease in the T_g_ of CLiPS, as depicted in Supplementary Fig. 3b. In this regard, we may conclude that a lower Cl groups content is required to ensure a fast self-healing speed. In contrast, this reduces the number of ionic trap sites that are required to enable mechanosensitive piezo-ionic dynamics. To ensure sufficient trap sites for excellent ion trapping along with fast self-healing speed in the CLiPS, an optimum condition was obtained with the CLPU@E5 sample together with a 30 wt% IL (see Supplementary Note 1, Supplementary Figs. 4–7, and the experimental section for the optimization approach adopted to obtain the optimum concentration of IL and preparation of the CLiPS).

### Ultrafast self-healing mechanism of both CLiPS and electrodes

**Fig. 2:**
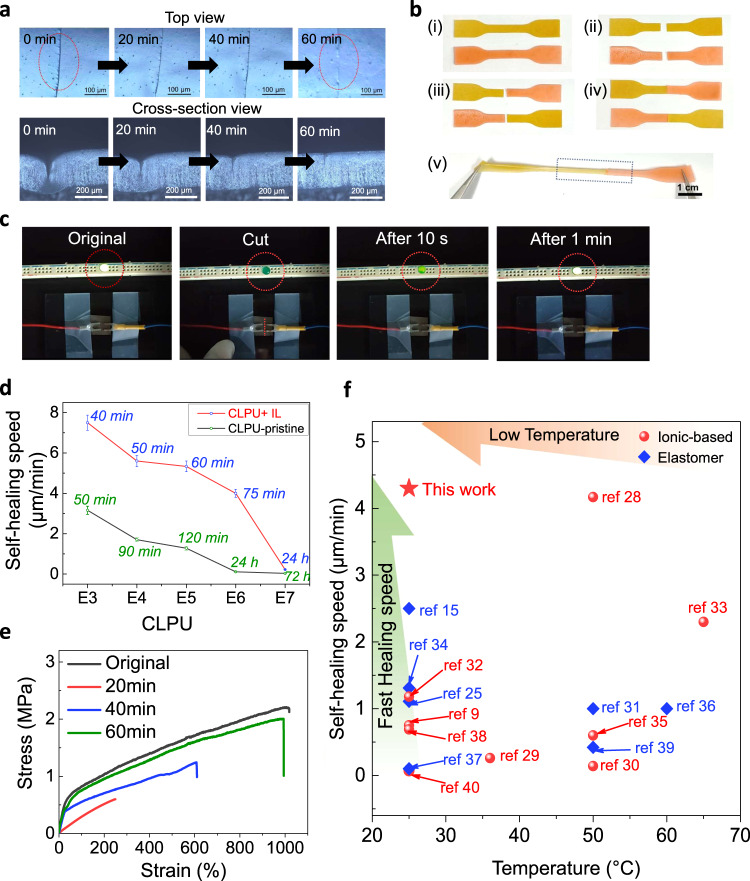
Autonomous self-healing demonstration of the CLiPS device. **a** Autonomous self-healing of a scar within 60 min at room temperature (RH 20–40%) observed via an optical microscope. The experiments (top; scar bar 100 µm and cross-section view; scar bar 200 µm) were conducted independently and produced similar results. **b** Two cut and spliced individual CLiPS films healed together to withstand stretching. **c** Demonstration of the tractive self-healing of the electrode’s conductivity using an LED. **d** Comparison of the self-healing speeds as a function of Cl groups concentration. E3 and E7 have the lowest and highest Cl groups content, respectively. CLPU-IL and CLPU-pristine denote CLPU films with 30 wt% IL and without IL concentration, respectively. All self-healing tests were carried out at room temperature (RH 20–40%) without external stimuli. **e** Stress–strain curves of the original and self-healed films at various healing times. **f** Comparison of self-healing speeds between CLiPS and other room-temperature self-healing dielectric elastomers (blue color) and ionic-based materials (red color).

Owing to the molecular nature of the CLPU, the CLiPS-based piezocapacitive device exhibits a fast self-healing speed even at room temperature without external stimuli. We note that the structure of the IPDI substantially contributes to the self-healing efficiency of the CLiPS by providing a high polymer chain mobility to enhance the activation of dynamic exchange reactions of disulfide bonds while retaining its tough properties^15,23,24^. Owing to the combined toughness properties of the IPDI and the Cl groups, the CLiPS exhibited excellent elastic recovery (100%) without any constraints (Supplementary Fig. 8). The disulfide bonds utilized in this study are well-known reversible dynamic bonds exhibiting effective exchange reactions that can be activated at lower temperatures. When damaged, these disulfide bonds undergo metathesis reaction mechanisms to repair themselves autonomously^15,25^. The combined effects of high chain mobility in the IPDI, abundant reversible disulfide bonds, as well as the plasticization effect of the IL, drive the fast autonomous self-healing of the CLiPS. As illustrated in Fig. 2a and Supplementary Movie 1, a scratch test was performed under a real-time optical microscope. The top and cross-section views (analysis conducted independently) revealed consistency in their results, where the scar on the CLiPS film disappeared within 60 min at room temperature (relative humidity, RH 20–40%). Similarly, Fig. 2b depicts the complete cut scenario of two individual CLiPS pieces, which were then healed together under the same conditions to withstand stretching (samples were colored for clearer experimental demonstration).

To realize self-healing of the entire device architecture, including the electrodes and interfaces, the same polymer composition was employed to fabricate the electrodes. Fig. 2c and Supplementary Movie 2 illustrate the self-healing of the electrodes, which consist of an Ag nanowires (AgNWs) percolation network on the surface of CLPU@E5 (see the experimental section for details on the fabrication methods). The electrodes achieved tractive self-healing owing to the tractive force generated by the self-healing driving force of the CLPU@E5 films connecting the cracked surfaces^26,27^. To confirm the healing property, the device was connected to a light-emitting diode (LED). After cutting across the width with a sharp blade, the AgNWs were electrically insulated from each other, thereby causing the LED intensity to dim completely. Within 1 min, the brightness was restored confirming the self-healing of the electrodes. The healing process can be attributed to the self-healing of the CLPU@E5 film, as well as the fusion of AgNWs to reconnect the percolation network over the crack surface^27^. The fast self-healing recorded here can be attributed to the fact that only approximately one-third of the total film thickness was cut, allowing the percolation network to quickly reestablish connection because the lowermost part of the film was still unscathed. If the film was cut through completely, it would be impossible to achieve perfect alignment of the AgNW networks that strongly affect the restoration of the conductive surface^27^.

Figure 2d compares the self-healing speeds of the CLPUs with or without IL concentration as a function of Cl groups content. As discussed previously, we note that the plasticization effect of the IL enhanced the polymer chain mobility, which produced faster self-healing speeds in the CLiPS than in the pristine materials. To affirm the strong autonomous self-healing efficiency of the CLiPS, stress–strain tests were performed on the healed samples at various time intervals. Figure 2e and Supplementary Table 4 present the mechanical properties of the original and healed films at different time intervals, demonstrating a maximum self-healing efficiency of 91% within 60 min. In addition, as presented in Fig. 2f, CLiPS exhibits fast self-healing speed of 4.3 µm/min as compared with other room-temperature self-healing dielectric elastomers and ionic-based materials with different healing mechanisms, including imine bonds, metal coordination, hydrogen bonds, disulfide bonds, electrostatic interactions, and their combinations^9,15,25,28–40^. The proposed CLiPS demonstrated superior performance in terms of the significant metrics in self-healing evaluation: healing speed (4.3 µm/min) and autonomous driving force.

### Molecular characterization and complex impedance behavior

**Fig. 3:**
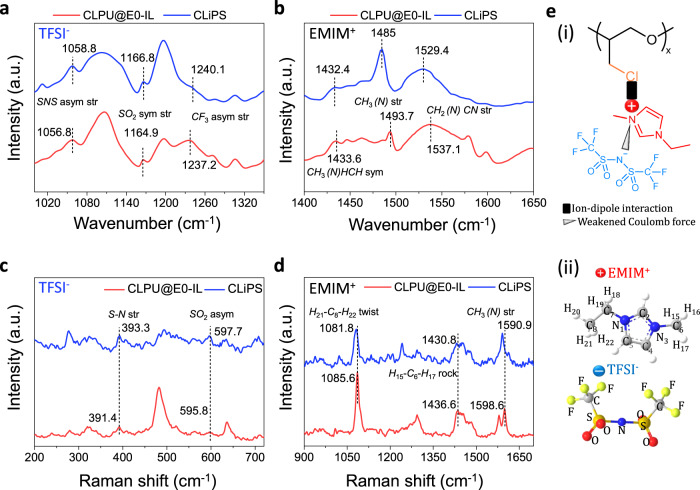
Molecular characterization of CLiPS. ATR-FTIR spectra of CLPU@E0-IL (used as reference) and CLiPS films. ATR-FTIR spectra in the spectral regions of **a**, 1000–1350 cm^−1^ (pertaining to TFSI^−^ stretching) and **b**, 1400–1650 cm^−1^ (pertaining to EMIM^+^ stretching). **c**, **d** Raman spectra in the ranges of 200–720 cm^−1^ and 900–1700 cm^−1^ corresponding to TFSI^-^ and EMIM^+^ vibrational bands, respectively. These spectra confirm the trapping of ion pairs to Cl groups via ion–dipole and Coulombic interactions. **e** (i) Schematic presenting of ion–dipole interaction between Cl groups and the [EMIM]^+^ cation, and Coulomb force between ion pairs, (ii) atomic number scheme of [EMIM]^+^ utilized in (**d**), and atomic structure of [TFSI]^−^.

To investigate the ion–dipole interactions between the Cl groups and the [EMIM]^+^[TFSI]^–^ ion pairs, attenuated total reflection–Fourier transform infrared (ATR-FTIR) analytical characterizations were performed on the IL, CLiPS, and CLPU@E5 (used as a reference). The spectra band at 1346 cm^−1^ representing *SO*_2_ antisymmetric stretching of the [TFSI]^–^ shifted to 1352 cm^−1^, whereas the 1051 cm^−1^ spectra band attributed to *N–S* antisymmetric stretching shifted to 1058 cm^−1^, as depicted in Supplementary Fig. 9a, b^35,41^. These shifts indicated that the Coulomb interactions between the ion pairs weakened as the [EMIM]^+^ cations were dragged, owing to the strong pull exerted by the C–Cl dipoles in the CLiPS^41^. We note that these bonds stretching can provide strong evidence supporting the ion–dipole interactions generated between the Cl groups and the [EMIM]^+^ cations. Next, to confirm the hypothesis that the ions are trapped to the Cl groups, molecular characterizations of CLPU@E0-IL (with a 30 wt% IL, without Cl groups; used as a control sample) and CLiPS were further analyzed. Notably, the highly electronegative Cl atoms exert a strong electrostatic force on the positive ions, resulting in dragging of the [EMIM]^+^ cations, weakening the Coulombic force between the ion pairs. This generates extra freedom that enhances the dissociation and dispersion of the ionic species. This postulation is supported by the shifting of the FTIR vibrational bands corresponding to the *SNS*, *SO*_2_, and *CF*_3_^42^ bonds of [TFSI]^–^ (spectra range of 1000–1350 cm^−1^, Fig. 3a) toward higher wavenumbers. This indicates the extra freedom of the [TFSI]^–^ ions, caused by the pulling away of the cations via ion–dipole interactions with the C–Cl groups^35^. Correspondingly, Fig. 3b presents a comparatively large shift of the bands corresponding to the [EMIM]^+^ vibrational bands (*CH*_3_*(N)HCN* stretching, *CH*_3_*(N)* stretching, and *CH*_2_*(N)CN* stretching^42^ in the spectra region 1400–1650 cm^−1^) toward lower wavenumbers, clearly revealing the strong immobilization of the [EMIM]^+^ cations to the C–Cl groups. Complementary to the FTIR features, the Raman vibrational modes also confirm the confinement of the [EMIM]^+^[TFSI]^–^ ion pairs to the Cl groups. The relative shifting of the [TFSI]^–^ Raman vibrational modes corresponding to *S–N* stretching and *SO*_2_ asymmetric stretching^12,42^ (Fig. 3c) indicates a weakened interaction between the ion pair species owing to the strong pull on [EMIM]^+^ exerted by the dipoles. In addition, the considerably large shift of the [EMIM]^+^ Raman vibrational modes (*H*_21_–*C*_8_–*H*_22_ twist, *H*_15_–*C*_6_–*H*_17_ rock vibration, and *CH*_3_*(N)* stretching)^42,43^ (Fig. 3d) toward lower wavenumbers strongly support the trapping of [EMIM]^+^ in the CLiPS. The ion–dipole interactions between the [EMIM]^+^ cations and the Cl groups in the matrix also promote the uniform dispersion and self-dissociation of ion pairs throughout the CLiPS polymer matrix^44^ (Fig. 3e (i) and field emission scanning electron microscopy image in Supplementary Fig. 10a). In contrast, CLPU@E0-IL (without Cl groups) exhibits undissociated ion pairs due to the low polarity of the CLPU@E0 matrix, which leads to the formation of ion aggregations (Supplementary Fig. 10b)^45,46^.

**Fig. 4:**
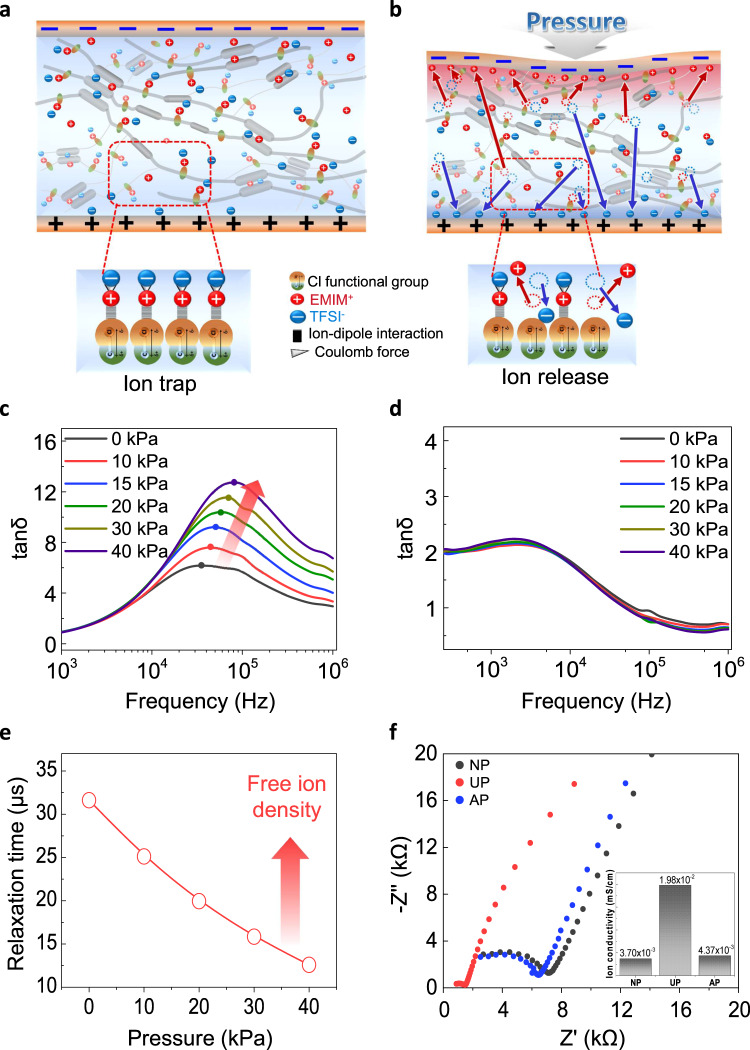
Molecular design and working principle of the trap and release-based CLiPS device. Design of the piezocapacitive device consisting of the CLiPS film sandwiched between AgNW/CLPU@E5 flexible electrodes (1 mV to 1 V). **a** Confinement of [EMIM]^+^[TFSI]^-^ ion pairs to Cl groups (trapped state) at a pre-stimulus condition. **b** Schematic of CLiPS demonstrating the pumping of ions owing to pressure-impelled breaking of ion–dipole interactions under deformation and EDL formation at the CLiPS/electrode interface. Ion dynamics and free ion density of (**c**) CLiPS and (**d**) CLPU@E0-IL films with a stepwise pressure increase. CLiPS exhibits trap and release mechanism as the free ion density increases with pressure. **e** Charge relaxation time decreases with increased pressure input owing to the release of more free ions in the CLiPS. **f** Nyquist plot of CLiPS under no pressure (NP), under pressure (UP), and after removing pressure (AP), confirming reversible movement of ions (insert shows ion conductivity under each condition).

Figure 4a illustrates a schematic structure describing the piezocapacitive pressure-sensing mechanism (termed mechanosensitive piezo-ionic dynamics) of CLiPS-based e-skin devices (1 cm × 1 cm, the thickness of 260 µm) sandwiched between two deformable self-healing electrodes (1.5 cm × 1.5 cm), creating electrical double layer (EDL) at the interface. At pre-stimulus conditions, the majority of the [EMIM]^+^[TFSI]^–^ ion pairs are trapped to the Cl groups within the PU matrix. We note that Cl groups are partially negatively charged species that attract positively charged [EMIM]^+^ cations (bounded to [TFSI]^–^ anions through Coulomb attractive forces^4^) via ion–dipole interactions. Nevertheless, some ions can exist independently across the polymer matrix, primarily through intercalation between the PU hard segments^3^ as depicted in Supplementary Fig. 5 (see Supplementary Note 1 for details). Under external pressure (Fig. 4b), the CLiPS-based e-skin underwent gradual deformation, thereby decreasing the distance between the top and bottom electrodes, which created a strong electric field. Notably, the deformation caused by the applied pressure initiates an ion-pumping phenomenon, releasing more free ions, which induces the formation of EDL at the electrode/electrolyte interface. The mechanosensitive piezo-ionic dynamics arise from the pressure-mediated breaking of the ion–dipole interactions, which causes the detachment of the ion pairs from the Cl groups to form the EDL. The established trap and release phenomenon of the ions ensures a low initial capacitance value, and alternatively, generates a high final capacitance value under pressure owing to the effective pumping of ions. This provides an improved control of piezo-ionic dynamics, in addition to providing high sensitivity, and high signal-to-noise level in the CLiPS-based e-skin devices.

In addition, the relationship between the trap and release mechanism of the ions and complex impedance behavior was investigated by performing stress relaxation tests on CLiPS and CLPU@E0-IL (used as reference) to analyze the free ion density in terms of applied pressure (see Supplementary Note 2 for more details). Generally, in the presence of an alternating current (AC) potential, mobile ions migrate toward the respective opposite electric charges, which leads to the formation of interfacial electrode polarization. The accumulation of mobile charges near the electrodes in the formation of the EDL, establishes the Debye length (λ_D_ or κ^−1^), which characterizes the distance to which the charged surfaces are screened by the ionic clouds in the diffusion layer from the bulk electrolyte^47,48^. According to the Debye–Falkenhagen model, the Debye length is inversely proportional to the ionic strength^47^, therefore, a higher free ion density decreases the Debye length. Subsequently, the timescale at which the ion dynamics become diffusive in a parallel RC (bulk resistance; *R* = *d/σA*, bulk capacitance; *C* = *∈A/d*) circuit is known as the charge relaxation time (*τ* = *∈/σ* = *RC*), and the crossover frequency of the imaginary and the real impedance in the Bode plot is referred to as the charge relaxation frequency (τ^–1^)^49^. It is therefore expected that with a stepwise increase in the pressure, the entire impedance plot should shift downwards due to the decreasing impedance and subsequent increase in the free ion density, as depicted in Supplementary Fig. 11a, for the CLiPS-based e-skin devices. However, the CLPU@E0-IL (without Cl groups)-based e-skin (Supplementary Fig. 11b) exhibits no significant change in the impedance plot. This is because the ions are not initially trapped, and it is difficult to expect pressure-mediated ion pumping for the release of ions under pressure. In addition, the frequency dependence of tan δ (Ɛ”/Ɛ’) can be utilized to explore the free ion number density and diffusivity by analyzing the shift in the relaxation peaks^50,51^. As depicted in Fig. 4c, τ^–1^ shifts toward higher frequencies as the pressure increases owing to the faster ionic atmosphere relaxation resulting from the enhancement in the free mobile ion diffusivity under pressure. In contrast, τ^–1^ in the CLPU@E0-IL (Fig. 4d) exhibits no evident shift because ions are not released with the increase in pressure, as the majority of the ion pairs already exist within the free volume of the polymer matrix (not bounded to PU chains)^3^. Hence, the ionic strength increases as more ions are released with increasing pressure in the CLiPS-based e-skin devices, which subsequently shortens the Debye length (high average free ion density in the diffusion layer) and generates lower relaxation time at higher pressures, as illustrated in Fig. 4e^48^. Furthermore, to explore the reversibility of ion movement, electrochemical impedance spectroscopy (EIS) Nyquist plots presented in Fig. 4f, were obtained for the CLiPS-based e-skin devices under the following conditions: no pressure (NP), under pressure (UP), and after removing pressure (AP). The results obtained clearly demonstrated the superb recovery of ion mobility after the removal of pressure, which confirmed the efficient reversible ion-pumping process in CLiPS. In contrast, no significant reversible ion-pumping process was observed in the CLPU@E0-IL as the majority of the ion pairs exist within the free volume of the polymer matrix (Supplementary Fig. 12).

### Piezocapacitive tactile sensing performance of CLiPS

**Fig. 5:**
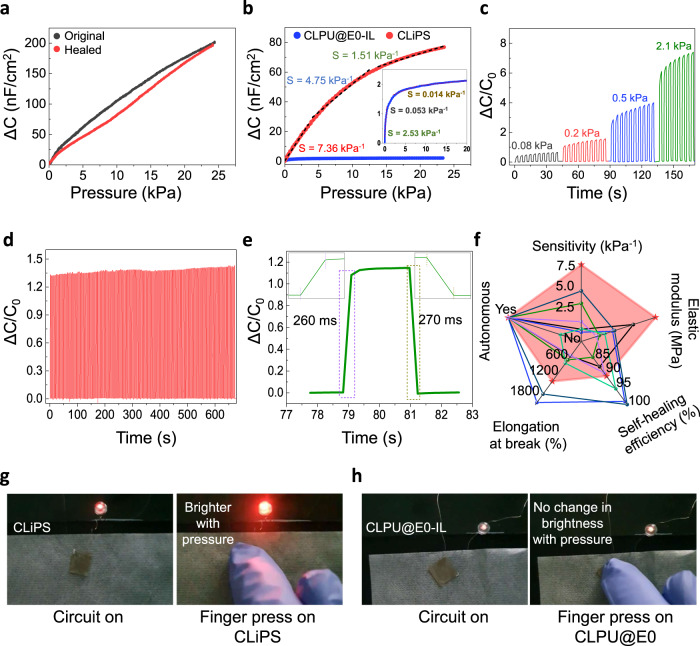
Sensing performance and practical application of the CLiPS device. **a** Pressure response of the original and self-healed films under a static pressure condition (1 V applied bias @ 100 Hz). **b** Pressure sensitivity comparison between CLiPS and CLPU@E0-IL (insert, black) (used as a reference), applied bias of 100 mV at 20 Hz. **c** Relative change in capacitance plots as a function of the applied pressures (0.08 kPa, 0.2 kPa, 0.5 kPa, and 2.1 kPa) with respect to time (applied bias voltage of 100 V at 100 Hz). **d** Mechanical durability test results of the CLiPS-based sensor (250 cycles), applied bias at 100 mV at 100 Hz). **e** Transient response time of the CLiPS piezocapacitive sensor at a loading pressure of 90 Pa. The insert represents a response time of 260 ms and a reset time of 270 ms. **f** Comparison between CLiPS-based sensor and previously reported autonomous self-healing iontronic pressure sensors in terms of autonomous capability, sensitivity, modulus, self-healing efficiency, and stretchability. Red, black, blue, green, dark-blue, violet, and turquoise colors correspond to this work, ref. 40, ref. 32, ref. 38, ref. 52, ref. 53, and ref. 33, respectively (see detail on Supplementary Table 5). **g**, **h** Photographs showing LED brightness before and after applied pressure of the CLiPS-based device (left) and the CLPU@E0-IL-based device (right).

Next, we confirmed the pressure-sensing response of self-healed CLiPS as compared to the original under static pressures. The obtained pressure responses strongly confirm retention of the sensing property even after self-healing (Fig. 5a). The piezocapacitive pressure-sensing capability of CLiPS-based e-skin devices under different applied bias voltages (1 mV, 100 mV, and 1 V) at different frequencies (1 kHz, 100 Hz, and 20 Hz) was also confirmed (Supplementary Fig. 13). Moreover, the pressure response of various CLiPS (10–40 wt%)-based sensors were also investigated. As discussed previously, with an increasing pressure input applied to the CLiPS-based devices, numerous mobile ions (initially trapped) are released to the interface between the CLiPS and the electrodes, which induces EDL formation. Furthermore, with the increase in IL concentration, the capacitance change increased tremendously owing to the existence of more free mobile ions (Supplementary Fig. 14). The high capacitance observed at a low frequency of 20 Hz results from effective EDL relaxation, whereas the increase in the AC bias frequency drastically decreases the capacitance owing to the limitation of the EDL relaxation time. In addition, the Cl groups also contributed to the overall higher capacitance of the CLiPS-based devices owing to the enhanced dielectric constant (Ɛ’) of CLiPS (further discussion below). The EDL formation phenomenon can be further enhanced under high voltage bias conditions, as explained in Supplementary Note 3. The pressure sensitivity, defined as *S* = *δ*(*ΔC/C*_*0*_)*/δP*, (where *C*_*0*_ denotes capacitance value without applied pressure, *ΔC* denotes change in capacitance, and *P* denotes applied pressure) of the CLiPS (S = 7.36 kPa^−1^–1.51 kPa^−1^) is substantially much higher compared to that for the CLPU@E0-IL (used as reference) (S = 2.53 kPa^−1^–0.014 kPa^−1^) (Fig. 5b). The sensitivity of CLPU@E0-IL over 10 kPa portrayed a typical saturation behavior, whereas CLiPS demonstrated remarkable sensitivity over the same range. This improved sensitivity of CLiPS can be directly attributed to the Cl groups–induced effective piezo-ionic dynamics, resulting in high *C*_*p*_*/C*_*0*_ values. The time-dependent response and recovery of the CLiPS-based device were analyzed under dynamic pressure conditions. As depicted in Fig. 5c, an excellent reliable and repeatable capacitance change with a dynamically increasing applied pressure was demonstrated. Moreover, continuous loading–unloading cyclic tests were performed to characterize its durability (Fig. 5d). The highly reproduced capacitance signals confirmed the phenomenal durability and structural integrity of the CLiPS-based e-skin device. It also demonstrated an excellent response and reset time, as depicted in Fig. 5e. To represent the strengths of the CLiPS-based device, this study is compared with previously reported autonomous self-healing iontronic pressure sensors. As presented in Fig. 5f, the CLiPS-based device demonstrates superior features, such as higher sensitivity and superior elastic modulus, compared to other recently reported self-healing iontronic pressure sensors^32,33,38,40,52,53^.

Furthermore, to investigate the impact of Cl groups on the capacitance of CLiPS, the dielectric behavior, the initial and final capacitance values of films with different amounts of Cl groups were analyzed. We note that the introduction of various concentrations of Cl groups contributed enormously to the capacitance values of CLiPS (Supplementary Fig. 15) owing to the increased dielectric constants (Ɛ’) of the CLPUs. This phenomenon occurred because the chlorinated soft segment chain considerably enhanced the polarity of the CLPUs through the induced dipole moment in the polymer matrix, generating a higher dielectric constant^54^. The dielectric constants of individual films without IL concentrations (CLPU-pristine) were calculated based on data obtained from EIS^51^. The dielectric constants increased as the Cl groups content increased, with CLPU@E0 (without Cl groups and no IL) demonstrating the lowest dielectric constant value (Supplementary Fig. 16a). In addition, Supplementary Fig. 16b also presents the maximum capacitance values of the CLPU-pristine films (the area and thickness of films were kept constant), which evidently depict an increasing trend as the Cl groups content increased. This trend is directly associated with an increase in the dielectric constant with the introduction of more Cl groups. Hence, with the addition of 30 wt% IL to the CLPUs, the initial capacitance values increased as more Cl groups were introduced owing to the steady enhancement in the dielectric constant of the successive samples (Supplementary Fig. 17a). Subsequently, under pressure (~23 kPa^−1^), the final capacitance of the samples increased with the Cl groups content, owing to the release of more ions that were initially trapped, as clearly depicted in Supplementary Fig. 17b. These results both affirm the increase in polarity (dielectric constant) with the introduction of more Cl groups and strongly support the effective trap and release concept in CLiPS.

As a demonstration, the CLiPS-based e-skin device was used as a pressure-induced tactile sensor to modulate the brightness of an LED. The combination of the high-pressure sensitivity, wide dynamic pressure-sensing range, and superior elastic recovery enabled CLiPS to function as a capacitive-based tactile sensor. The CLiPS-based device functioning as a variable capacitor was connected in series to an LED using an AC power source (circuit configuration depicted in Supplementary Fig. 18). When a human finger contacted the CLiPS-based device and pressure was applied, the intensity of the red LED increased (Supplementary Movie 3) as the bulk resistance decreased with the applied pressure (Fig. 5g). The explanation for the increased LED intensity is based on two concepts. (1) The capacitive coupling of the AC bias drives the LED, where a simple circuit of capacitor and LED in series generally suffices because the *τ* = *RC* timescale is short compared to the AC power timescale^55^. Therefore, the current flow is governed by the capacitive impedance (reactance) */Z/* = *1/ωC*, where *ω* denotes the angular frequency of the AC power source, *C* denotes the capacitance of the device, characterized by *V(t)* *=* *V*_*0*_ *sin(ωt)*, and *V*_*0*_ denotes the peak voltage^55^. (2) CLiPS functions as an ionic conductive matrix, which induces electrode polarization under an applied AC bias, allowing charges to flow through the device. Before applying pressure using a finger, a few mobile ions are available for charge flow. When pressure is applied by a finger, the pressure-mediated ion-pumping phenomenon in CLiPS releases more mobile ions, which decreases the bulk resistance and allows more charge to flow through to the LED, leading to a higher intensity. In contrast, the CLPU@E0-IL-based device (used as a reference) demonstrated no significant change in the LED intensity even when a high pressure is applied by the human finger (Fig. 5h and Supplementary Movie 4). This is because it is difficult for pressure-mediated ion pumping to release ions under pressure in CLPU@E0-IL because the ions are not initially trapped. Through this demonstration, we not only confirmed the pressure-sensing performance but also the effective piezo-ionic mechanism in CLiPS-based e-skin device.

## Discussion

Inspired by the self-healing properties and mechanosensory system of the human skin, we developed an autonomous self-healing CLiPS material from a homogeneous system with mechanosensitive piezo-ionic dynamics. We recorded an unprecedented self-healing speed (4.3 µm/min at 25 °C) and a tractive self-healing of electrodes within 1 min owing to the combined effect of high chain mobility IPDI, abundant reversible disulfide bonds, and the plasticization effect of IL. Notably, the key to realizing effective piezo-ionic dynamics in this system is the introduction of Cl groups into the PU matrix, which plays a pivotal role in the reversible ion–dipole bond-triggered ion-pumping process in CLiPS. This unique design approach enables the device to exhibit ion trap and release phenomena, which facilitate the realization of excellent pressure sensitivity without any surface modification of the active layer or electrodes. Furthermore, the high structural stability and reproducibility indicate that the CLiPS-based e-skin device could sense various pressures, thus validating its application for touch modulation in next-generation wearable technologies and smarter human–machine interfaces.

## Methods

### Fabrication of Cl-functionalized iontronic pressure sensor (CLiPS) film

The fabrication of the self-healing CLiPS film primarily involved (i) the synthesis of poly(epichlorohydrin-co-tetrahydrofuran) diol (PET), (ii) the synthesis of Cl-functionalized polyurethane (CLPU), and (iii) the preparation of a CLiPS film. The materials utilized were as follows: Bis(4-hydroxyphenyl) disulfide (98.0%) (BHPDS), epichlorohydrin (ECH) (99.0%), tetrahydrofuran (THF) (99.5%), and methylene chloride (MC) (99.5%) were purchased from SAMCHUN Chemical Co. (Korea). *N, N*-Dimethylformamide (DMF) (99.0%), boron trifluoride tetrahydrofuran complex (>99.5%), butane-1,4-diol (BDO) (99.0%), dibutyltin dilaurate (T-12) (95%), and isophorone diisocyanate (IPDI) (98.0%) were obtained from Sigma-Aldrich Chemical Co. (Korea). (i) The PET was synthesized via cationic ring-opening polymerization using a septum-capped reactor. The reactor was purged with N_2_ for 1 h and then a reaction of MC (270 g), BDO (5.96 ml), and boron trifluoride tetrahydrofuran complex (1.746 ml) was initiated for 20 min. The mixture of purified ECH (118 g) and THF (91.95 g) was then introduced dropwise through the septum for 4 h, and the reaction continued for another 2 h. The reaction was terminated after 20 ml of distilled water was added and stirred for 20 min. The mixture was extracted many times with water after dilution in MC. To remove the residue MC, the solution was evaporated, then dried under vacuum for 24 h at 60 °C^56^. using ECH and THF with a molar ratio of E3 (ECH 3:7 THF), E4 (ECH 4:6 THF), E5 (ECH 5:5 THF), E6 (ECH 6:4 THF), and E7 (ECH 7:3 THF) (structural representation in Supplementary Fig. 1a, b). MC (270 g), BDO (1,4-butanediol) (5.66 ml), and the boron trifluoride THF complex (1.75 ml) were added and stirred for 20 min. Then, the purified ECH (118 g) and THF (92 g) mixture was added via dropwise method over 4 h and allowed to react for 2 h. The reaction was terminated by adding 20 ml of distilled water and stirring for 20 min. The mixture was extracted several times and dried in vacuum at 80 °C for 24 h. (ii) The CLPU was synthesized at molar ratio of PET:BHPDS:IPDI = 8:1:9 using T-12 (dibutylin dilaurate) as the catalyst; the mixture was stirred at 60 °C using a magnetic stirrer at 80 °C under a nitrogen atmosphere. After the reaction, the mixture was precipitated using distilled water and dried. For the synthesis of various CLPU-pristine samples (film thickness of 154 ± 4 µm), the molar ratio of IPDI to BSOH were fixed at a constant value, whereas the molar ratio of PET was optimized as previously described. Samples E3 and E7 had the lowest and highest concentration of Cl groups, respectively. (iii) The CLiPS film was prepared by dissolving E5 (ECH 5:5 THF) and a 30 wt% [EMIM]^+^[TFSI]^–^ (1-Ethyl-3-methylimidazolium bis(trifluoromethylsulfonyl)imide, Sigma-Aldrich) in tetrachoroethlene (TCI Japan) and DMF (Sigma-Aldrich) at a mass ratio of 1:1 under stirring at room temperature for 8 h. To obtain a desirable thickness, a fixed amount was poured into a square Teflon mold and annealed at 80 °C for 48–72 h under optimized conditions (starting from 40 °C at 10 °C/ h temperature intervals). Similarly, CLPU@E0 and CLPU@E0-IL films (ECH 0:10 THF; samples without any Cl groups, Supplementary Fig. 1c) were also prepared under identical conditions as described above and used as references. Notably, in this study, the IL weight percentage represent the weight ratio of the IL to IL + PU. Therefore, CLPU@E0-IL contained 30 wt% IL + CLPU@E0.

### Fabrication of self-healing flexible electrodes

Flexible electrodes were prepared by spray coating an (SRC-200 VT, E-FLEX Korea, nozzle; 0.05 mm, pressure; 200 mbar) Ag nanowire (AgNW) solution onto a Teflon mold and heat treatment at 90 °C for 30 min to reduce the resistance of the inter-nanowires. The AgNW solution (4 g ml^–1^ in IPA) was prepared by diluting an AgNW suspension (Nanopyxis Corp., diameter: 32 ± 5 nm length: 25 ± 5 µm). A solution of CLPU@E5 was drop-cast onto the AgNW coating on the Teflon mold and then annealed under the optimized conditions described above. Therefore, the AgNW percolation network was transferred onto the CLPU@E5 film, which was peeled off from the Teflon mold to form AgNW/CLPU@E5 composite, and its conductivity was analyzed using a digital multimeter before further experiments. To connect with the measuring instrument, Ag wires (Nilaco Corp., diameter: 50 µm) were attached to the electrodes.

### Material characterization

The mechanical properties of the CLPU films were analyzed using a universal testing machine (UTM, Instron Instrument) with a strain rate of 5 mm min^–1^ using a 5 N load cell. All samples considered in this study were prepared according to ASTM standards (ASTM D638–5). The Young’s modulus was determined based on the slope of the stress–strain curves (0–5% strain values). Differential scanning calorimetry (DSC-Q20, TA instrument) was used to analyze the glass transition temperatures (T_g_) of the polymer films. The experiments were conducted under a nitrogen atmosphere over 1.5 heating and cooling cycles, and a scanning temperature range from −70 to 200 °C with a ramp rate of 5 °C min^–1^ (film sizes of ~7 mg). Field emission scanning electron microscopy (Thermo Scientific^TM^ SEM Verios G4 UC) equipped with an energy-dispersive X-ray system was used for surface morphological characteristics and line profile analysis. Attenuated total reflection–Fourier transform infrared (ATR-FTIR) spectroscopy spectra were obtained using a Bruker Optics GmbH (Germany) spectrometer in the attenuated total reflection (ATR) mode (ZnSe crystal). Each spectrum was obtained as the average of 64 scans (resolution of 2 cm^–1^) was recorded from 4000 to 600 cm^–1^. Raman spectroscopic measurements were conducted using a Thermo Scientific^TM^ DXR^TM^2 Raman microscope. The laser power was 10 mW (max) for 532 nm laser excitation with an exposure time of 0.01 s for 1000 scans. X-ray diffraction analysis was performed using a Bruker D8 Advance diffractometer with Cu-Kα radiation (wavelength = 1.54060 Å). Diffraction scans were performed at an angle of 10–90° 2θ, with a scan speed time of 0.32 s.

### Electrical characterization

Capacitance measurements were performed by sandwiching the CLiPS films (thickness ~260 µm, area 1 cm^2^) between two AgNW/CLPU@E5 electrodes in a piezocapacitive device configuration and connecting them to a precision LCR meter (Agilent Keysight Technologies, E4980A). To connect with the measuring instrument, Ag wires (Nilaco Corp., diameter: 50 µm) were attached to the electrodes. Notably, electrochemical impedance spectroscopy (EIS) is a powerful tool for investing the complex impedance behavior of mobile ions as well as ion-transport phenomena in polymer electrolytes and their electrode/electrolyte interfaces. Herein, EIS data were recorded at room temperature by using an electrochemical analyzer PGSTAT302N (Metrohm Autolab) in a 0.1 Hz–1 MHz frequency range with a 10 mV alternating current (AC) signal. To analyze the ionic conductivity, a coin cell (Hohsen Corp., Japan) was used for the EIS measurements of different films. Using the appropriate equivalent circuit models built in NOVA software (Metrohm Autolab), all the impedance spectra were fitted, and the bulk resistance (*R*_*b*_) of the devices was evaluated. The ionic conductivity was determined based on the bulk resistance values as ${{{{{\rm{\sigma }}}}}}=\left(\frac{l}{{R}_{b}\,{X\; A}}\right)$, where *σ* denotes ionic conductivity, *l* denotes the thickness of film, *R*_*b*_ denotes the bulk resistance from the EIS Nyquist plot and, *A* denotes he electrode area.

### Analysis of pressure response and sensitivity

To investigate the pressure response of the iontronic pressure sensors, a custom-built sensor probe station with a programmable *xy-* and *z-*axis stage (resolution of 0.1 µm) and a force gauge (Mark-10, resolution of 0.005 N) were utilized. The corresponding pressures were determined by dividing the load with the pressing dimensions of the unit film. In addition, the sensitivity of the piezocapacitive pressure sensor device for analysis of the diverse pressure spectra was determined based on the slope of the relative capacitance change versus pressure. For the in situ simultaneous recording of capacitance change and applied load, the measuring instrument was connected to a customized LabView-based program.

### Pressure-induced tactile sensor demonstration

The device demonstration was set-up in a circuit configuration, where the CLiPS-based device was connected in series to a light-emitting diode (LED). The specifications for the LED are as follows; brightness: 300–400 mcd; wavelength: 325–630 nm; diameter: 5 mm; voltage: 1.9–2.2 V; current: 5–20 mA. The CLiPS-based device was prepared with a top electrode/CLiPS/bottom electrode (Metal–Insulator–Metal) structure described in the previous sections, as depicted in Fig. 1b. A pulse generator (Keysight Technologies, 33500B series waveform generator) was used to supply AC power to activate the circuit.

## Supplementary information

Supplementary Information

Description of Additional Supplementary Files

Supplementary Movie 1

Supplementary Movie 2

Supplementary Movie 3

Supplementary Movie 4

## Data Availability

All relevant data supporting the results of this study are available within the article and its supplementary information files. Further data are available from the corresponding authors upon request.
