# Supplementary material for: Ultrafast, autonomous self-healable iontronic skin exhibiting piezo-ionic dynamics
Source: Nat Commun. 2022 Dec 13;13:7699. doi: 10.1038/s41467-022-35434-8 (PMC9744819; doi:10.1038/s41467-022-35434-8)
Supplement: Supplementary file 1 — Supplementary Information [file 41467_2022_35434_MOESM1_ESM.pdf]

Supplementary Information

Ultrafast, autonomous self-healable iontronic skin exhibiting piezo-ionic dynamics

Elvis K. Boahen[1]†, Baohai Pan[2]†, Hyukmin Kweon[1], Joo Sung Kim[1], Hanbin Choi[1], Zhengyang Kong[1], Dong Jun Kim[1], Jin Zhu[3], Wu Bin Ying[1,3,*], Kyung Jin Lee[2,*], and Do Hwan Kim[1,4,*]

[1] Department of Chemical Engineering, Hanyang University, Seoul 04763, Republic of Korea.

[2] Department of Chemical Engineering and Applied Chemistry, College of Engineering, Chungnam National University, 34134, Republic of Korea.

[3] Ningbo Institute of Materials Technology and Engineering, Chinese Academy of Sciences, Ningbo 315201, People's Republic of China.

[4] Institute of Nano Science and Technology, Hanyang University, Seoul 04763, Republic of Korea.

†These authors contributed equally to this work.

*Corresponding authors email: yingwubin@nimte.ac.cn (W.B.Y.); kjlee@cnu.ac.kr (K.J.L.); dhkim76@hanyang.ac.kr (D.H.K.)

This PDF file includes:

- Supplementary Notes 1 to 3
- Supplementary Figures 1 to 18
- Supplementary Tables 1 to 5
- Supplementary References 1 to 26

Supplementary Note 1: Synthesis, morphological characterization of CLPU film, and the optimization approach of CLiPS with various ionic liquid concentration (10–40 wt%).

The fabrication of the self-healing CLiPS primarily involves three steps (Supplementary Fig. 1 and Fig. 1c, Main manuscript) (i) the synthesis of poly(epichlorohydrin-co-tetrahydrofuran) diol (PET), (ii) the synthesis of Cl functionalized polyurethane (CLPU), and (iii) the preparation of a CLiPS film. Supplementary Fig. 1 presents the synthesis process of CLPU under optimized conditions. The PET was synthesized via cationic ring opening polymerization[1], as mentioned in the Method section (Main manuscript), using epichlorohydrin (ECH) and tetrahydrofuran (THF) to form the soft segment of the polyurethane (PU) structure. The ECH component contains the Cl functional groups (Cl groups), which is the key component in our PU structure for piezo-ionic dynamics.

Theoretically, all halogen groups can be utilized as trap sites to established ion trap and release phenomenon via ion-dipole interactions. Nevertheless, we introduced the Cl group among halogen ones owing to higher dipole moment in this work. In general, dipole moment is a product of charges and the distance between the charges in a molecule. The Cl group has been reported to generate the highest dipole moment, due to the longer distance and high partial charges between the C and Cl atoms[2,3]. Although the F group possesses superior charge (highest electronegativity), the distance between the C and F atoms are the lowest, thereby creating a lower dipole moment in the F group. In contast, the Br group possesses comparably longer distance between atoms due to the larger size of the Br atom. However, because it possesses a very small charge, it tends to exhibit a lower dipole moment. A lower dipole moment would correspondingly weaken the trapping effect on ions. Therefore, in this work, the Cl group was chosen because it exhibits the optimum conditions for higher dipole moment for the establishment of strong trapping effect on ions.

Through one-step polymerization method, CLPU samples were synthesized using PET, dynamic disulfide bonds (BHPDS), and isophorone diisocyanate (IPDI) at a molar ratio of PET:BHPDS:IPDI = 8:1:9 using dibutylin dilaurate as catalyst. The IPDI and the BHPDS constitute the hard segment, and the ECH constitutes the soft segment of the polyurethane, respectively. The hard segment can be defined as a chain segment formed by the reaction of diisocyanate with chain extender on the polyurethane main chain. Likewise, the soft segment can be defined as a chain segment consisting of oligomeric diols with glass transition temperature (T_g) below room temperature[4]. To establish the optimum content of Cl groups, various CLPU samples

were synthesized by optimizing the ratio of the ECH concentration in the soft segment with a molar ratio of E3 (ECH 3:7 THF), E4 (ECH 4:6 THF), E5 (ECH 5:5 THF), E6 (ECH 6:4 THF), and E7 (ECH 7:3 THF), where E3 and E7 have the lowest and highest Cl groups composition, respectively. We postulated that, more Cl groups composition would reduce the self-healing speed due to their intrinsic toughness properties, whereas increased Cl groups are necessary for better ion trapping effects for effective piezo-ionic dynamics. Hence, with a stepwise increase in the content of Cl groups, the self-healing speeds of E3, E4, E5, E6 and E7 were analyzed. Here, the self-healing speed (µm/min) can be defined as the speed taken by the damaged sample to attain maximum self-healing efficiency with or without external stimuli. Generally, the self-healing speed is calculated based on the ratio of [notch size] (µm) / self-healing time (min)[5,6]. However, in this work, the sample was cut completely with the knife piercing through to the lower part, hence, the notch size was taken as the thickness of the sample. Also, the self-healing efficiency was determined based on the ratio of (the stress of healed sample / the stress of original sample)[7] x 100%. The formula are as follows:

$$self - healing\ speed = \frac{Thickness\ (\mu m)}{Self - healing\ time\ (min)} \tag{1}$$

$$Self - healing\ efficiency = \frac{Stress_{healed}}{Stress_{Original}} \times 100\% \tag{2}$$

As postulated, there was a drastic reduction in the self-healing speed of the CLPUs as the content of Cl groups increased owing to the reduced polymer chain mobility (Supplementary Fig. 2 and Supplementary Fig. 3), with E3 and E7 exhibiting the highest and lowest self-healing speeds, respectively (Fig. 2d, Main manuscript**)**. Therefore, to ensure enough ion trap sites along with fast self-healing speed, CLPU@E5 exhibited optimum conditions.

For the fabrication of CLiPS, we explored the optimal concentration of IL for the maximum output performance and higher device sensitivity, by adding various IL concentrations (10, 20, 30, and 40 wt%) to CLPU@E5. Supplementary Fig. 4 presents the ion conductivity as a function of different IL concentration. The conductivity increased with IL concentration owing to the high presence of mobile ions. The peaks in the X-ray powder diffraction curves of the CLiPS (10-40 wt% IL) as depicted in Supplementary Fig. 5 reveal the amorphous nature of the films. The hike

in the intensity of low angle ($2\theta = 12.7$ °) with increasing IL content (prominent in 30 and 40 wt%) is as a result of intercalation of ions between the hard segments of PU matrix, often observed in ionic polymers[8]. Although there is plasticization effect of ions on the films, the intrinsic toughness properties of Cl groups in the soft segment coupled with the tough IPDI[9,10] within the hard segments retain the excellent mechanical properties of the CLiPS (Supplementary Fig. 6). To investigate the change in capacitance of these films, pressure dependent capacitance changes of the CLiPS with varying IL concentrations were performed. Supplementary Fig. 7 presents the initial (C_0) and final capacitance (C_p) values of CLiPS as a function of IL concentration under external mechanical pressure (0 ~ 23 kPa). The film with 30 wt% IL exhibits the highest C_p/C_0, which results in the maximum output sensitivity. Therefore, 30 wt% IL concentration was chosen as the optimum condition for further device analysis. As mentioned earlier, Cl groups possess dipole moment that interacts with the $[EMIM]^+$ cation via ion-dipole interactions, which results in the trapping of ion pairs. Attenuated total reflection-Fourier transform infrared (ATR-FTIR) spectroscopy was performed to analysis the ion-dipole interactions in CLiPS. The shifting of $TFSI^-$ FTIR spectra bands (Supplementary Fig. 9a, b), towards higher wavenumbers in CLiPS (compared to pure IL), confirms the ion-dipole interactions as the Coulomb forces between the ion pairs were weakened owing to the strong pull on the cations by the C–Cl dipoles (Fig. 3e (i)), which agrees with previous reports.[11,12]

Supplementary Note 2: Electrical characterization and piezo-ionic dynamics of CLiPS film

An investigation of the electrical characteristics and complex impedance behavior under applied external pressure can play a pivotal role to clarify the piezo-ionic dynamics in CLiPS-based e-skin device (Fig. 4c-e, Main manuscript and Supplementary Fig. 11). Notably, electrochemical impedance spectroscopy (EIS) is a powerful tool for investigating the complex impedance behavior of mobile ions as well as ion-transport phenomena in polymer electrolytes and their electrode/electrolyte interfaces. Herein, EIS data was recorded at room temperature by using an electrochemical analyzer PGSTAT302N (Metrohm Autolab) in a 0.1 Hz–1 MHz frequency range with a 10 mV alternating current (AC) signal. To analyze the ionic conductivity, a coin cell (Hohsen Corp., Japan) was used for the EIS measurements of different films (~260 µm). Using the appropriate equivalent circuit models built in NOVA software (Metrohm Autolab), all the impedance spectra were fitted, and the bulk resistance (R_b) of the devices was evaluated.

To support the hypothesis that $[EMIM]^+[TFSI]^-$ ion pairs are trapped to the Cl groups, we explored the relationship between piezo-ionic dynamics and the complex impedance behavior, by performing stress relaxation tests on CLiPS and CLPU@E0-IL (without Cl groups, used as reference), under applied external pressure conditions. For non-Faradaic or capacitive processes, ion migration and subsequent interfacial electrode polarization, occur in the presence of applied AC bias, where electrode polarization analysis can be utilized to investigate free-ion diffusivity and number density in ionic conducting materials. Under an applied electric field, mobile ions migrate and accumulate near the electrodes resulting in space-charge polarization, which results in the electric double layer (EDL) formation[13]. The total free ion number density can be related by the Debye length (λ_D *or* κ^{-1}), which characterizes the distance to which the charged surfaces are screened by ionic clouds in the diffusion layer from the bulk electrolyte[14,15]. The Debye length is related to the ionic strength according to the Debye-Falkenhagen model[14,16] as follows:

$$\frac{1}{D}\frac{\partial \rho}{\partial t} = (\nabla^2 - k^2)\rho \qquad (3)$$

where D is the diffusion coefficient, ρ is the charge density, κ^{-1} is the Debye length.

$$\kappa^{-1} = \sqrt{\frac{\epsilon_r \epsilon_0 \, k_B T}{2 N_A e^2 I}} \qquad (4)$$

$$I = \frac{1}{2}\sum_{i=1}^{n} c_i z_i^2 \qquad (5)$$

where ϵ_r is dielectric constant of electrolyte, ϵ_0 permittivity of free space, k_B Boltzmann's constant, T temperature, N_A Avogadro constant, I the ionic strength, c_i ion concentration and z_i is ionic charge. From these expressions, the Debye length is inversely proportional to the ionic strength. Therefore, the Debye length decreases with higher number of free ion density.

Herein, our system function similar to the RC (bulk resistance; $R = d/\sigma A$, bulk capacitance; $C = \epsilon A/d$) circuit where the time scale at which the ion dynamics become diffusive is known as the charge relaxation time ($\tau = \epsilon/\sigma = RC$) and the crossover frequency of the imaginary and the real impedance in the Bode plot is referred to as the charge relaxation frequency (τ^{-1})[17]. It is

therefore expected that the impedance from R and C decreases with an increase in pressure. We note that the CLiPS-based e-skin device exhibited a distinguishable decreasing impedance plot owing to the released of more mobile ions under increasing pressure (Supplementary Fig. 11a). In contrast, the CLPU@E0-IL-based device exhibits no significant change in impedance plot. This is because the ions are not initially trapped, and it is difficult to expect pressure-mediated ion pumping for the release of ions under pressure (Supplementary Fig. 11b). In addition, as the concentration of free ion number density increases, the electrode polarization would shift toward a higher frequency regime[16]. The frequency dependence of tan δ (Ɛ''/Ɛ') can be utilized to explore the free ion number density and diffusivity by analyzing the shift in relaxation peaks corresponding to the angular frequency (ω_{max}), at which the full development of electrode polarization takes place[13,18]. With the increase in free mobile ion number density and diffusivity under increasing pressure, τ^{-1} in the CLiPS shifts toward higher frequencies owing to the faster ionic atmosphere relaxation (Fig. 4c, Main manuscript). However, τ^{-1} in the CLPU@E0-IL (Fig. 4d, Main manuscript) exhibits no evident shift because ions are not released with the increase in pressure, as the majority of the ion pairs already exist within the free volume of the matrix[8]. Therefore, the CLiPS-based device exhibits shorter Debye length (high average free ion density in the diffusion layer), which results in the shift of τ^{-1} towards higher frequencies (shorter times), signifying lower relaxation time at higher pressures (Fig. 4e, Main manuscript). From the maximum peak in tan δ, the relaxation time can be expressed as $1/\omega_{max}$.

Supplementary Note 3: Pressure response of CLiPS films under different applied bias voltages and bias frequencies

The pressure responses of different CLiPS (10-40 wt%)-based mechanosensitive pressure sensors as depicted in Supplementary Fig. 13 and Supplementary Fig. 14, under different applied bias voltages and bias frequencies, exhibit similar tendency of capacitance changes. This is because a continuous increase in external pressure leads to a steady increase in the device capacitance. This trend can be explained by the pressure-mediated breaking of ion-dipole interactions, which results in the release of more free mobile ions, enhancing the formation of EDL at the CLiPS/electrode interfaces. Furthermore, the applied bias voltage effect on increasing tendency of initial (C_0, capacitance under no pressure) and the final capacitance (C_p, capacitance under pressure of ~23 kPa), imply that intensified electric field across the CLiPS influenced the

device capacitance. This can be explained by the weakening of ion-dipole interactions with the increase in applied voltage bias[19]. This results in the voltage driven migration of ions[20] towards the corresponding electrodes, which enhances the EDL formation. The pressure response under different applied bias voltages evidently support that the pressure sensing mechanism in CLiPS can be attributed to the Cl groups-induced piezo-ionic dynamics. This subsequently, results in the pumping of $[EMIM]^+[TFSI]^-$ ions pairs from the trap state to the electrolyte/electrode interfaces.

Moreover, the pressure responses of various CLPUs (different Cl groups content) with constant IL concentration exhibited a trend increase of capacitance change as Cl groups content increased. This phenomenon occurred because the chlorinated soft segment chain considerably enhanced the polarity of the CLPUs through induced dipole moment. In addition, the introduction of more Cl groups increased the number of trap sites for excellent ion trapping effect, hence, more ions can be released under pressure which subsequently increased the capacitance change.

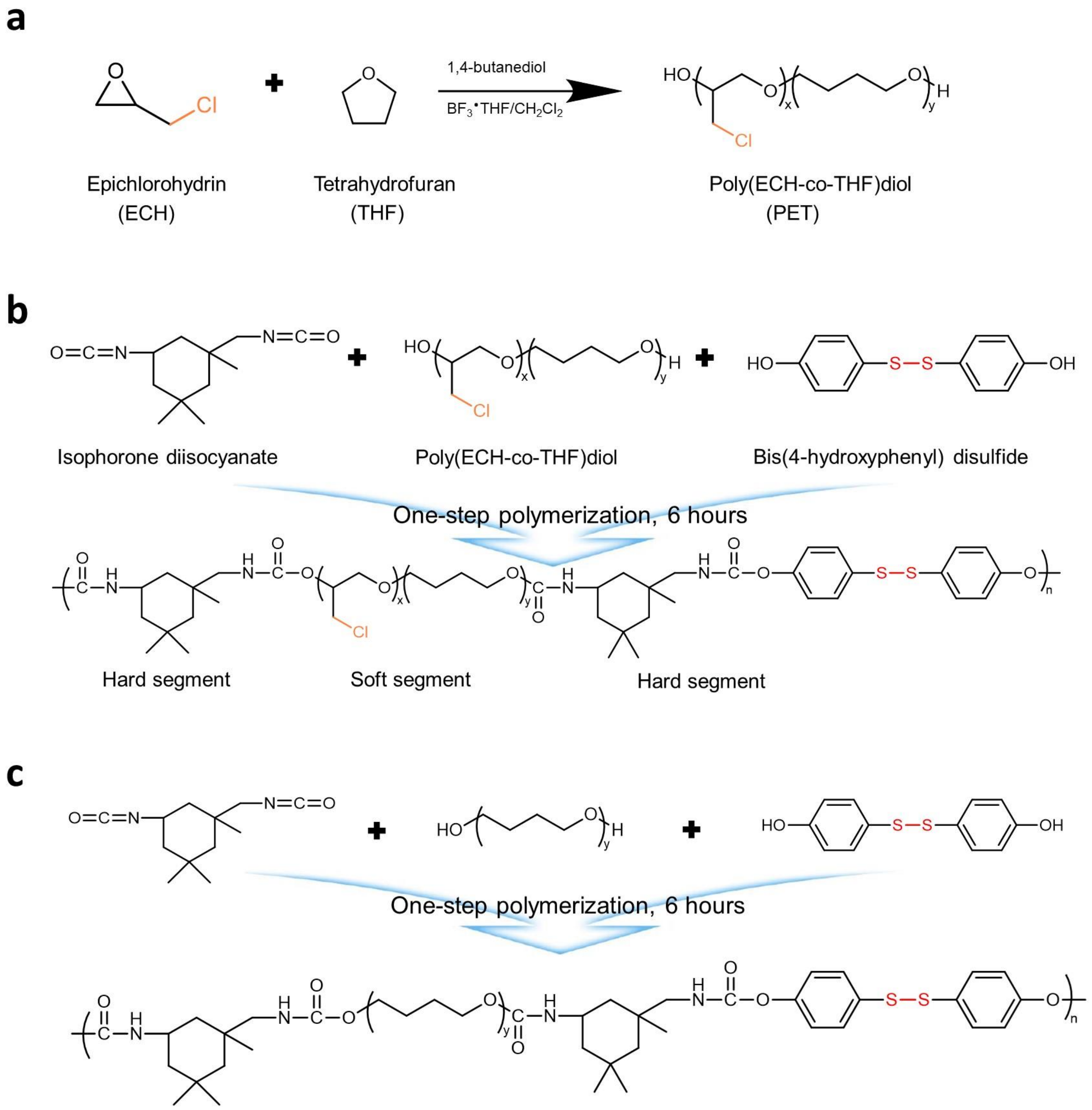

Supplementary Fig. 1 | Synthesis process of our polyurethane. Epichlorohydrin (ECH) molecule is composed of abundant Cl functional groups originating from the C-Cl bonds, which form the key component of the polyurethane. **a,** Synthesis process of Poly(ECH-co-THF) diol. **b,** Synthesis method of Cl functionalized polyurethane (CLPU). **c,** synthesis method of polyurethane without Cl groups (CLPU@E0) (used as reference).

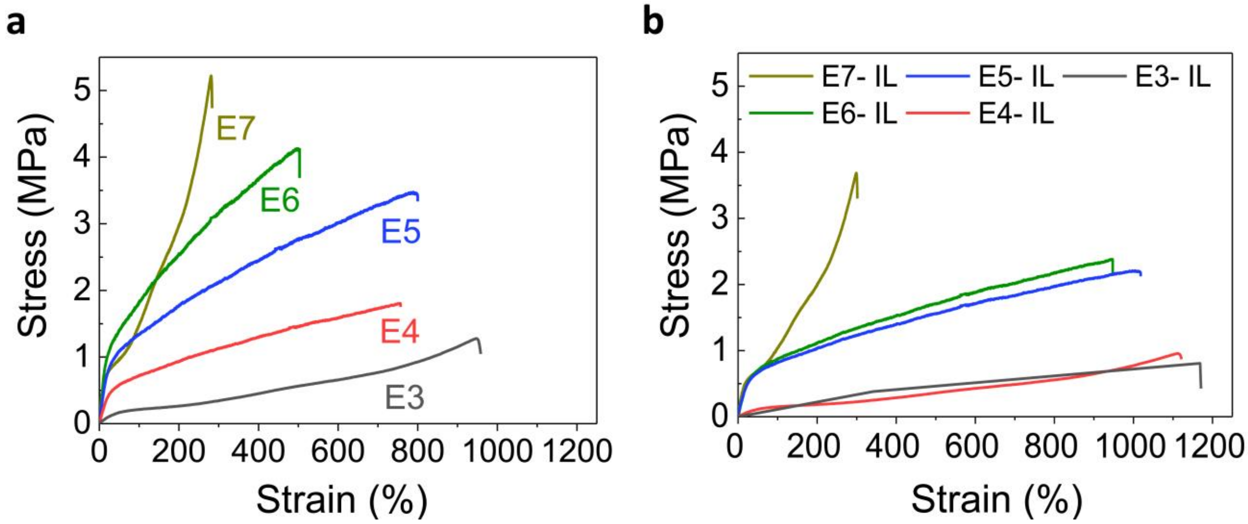

Supplementary Fig. 2 | Stress-strain curves of CLPU films in terms of Cl groups contents. a, Films without IL concentration (pristine). **b,** Films with 30 wt% IL concentration (CLPU-IL).

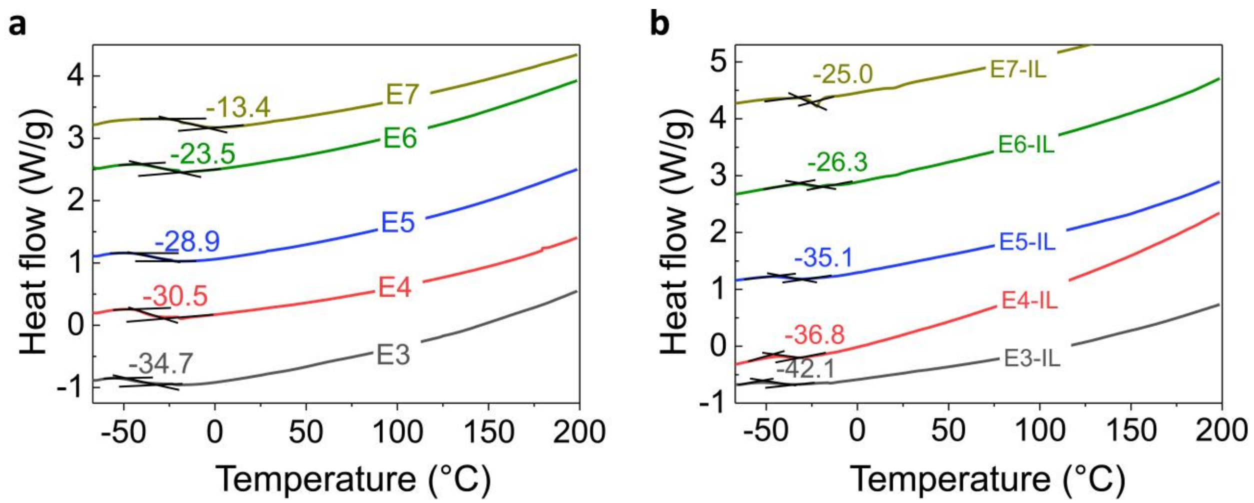

Supplementary Fig. 3 | DSC curves marked with glass transition temperature (T_g) of CLPUs. **a,** Pristine films (without IL) exhibiting a trend increase of T_g with the increase of Cl groups content. **b,** Films with 30 wt% IL concentration exhibiting a trend decrease of T_g confirming the plasticization effect of ions.

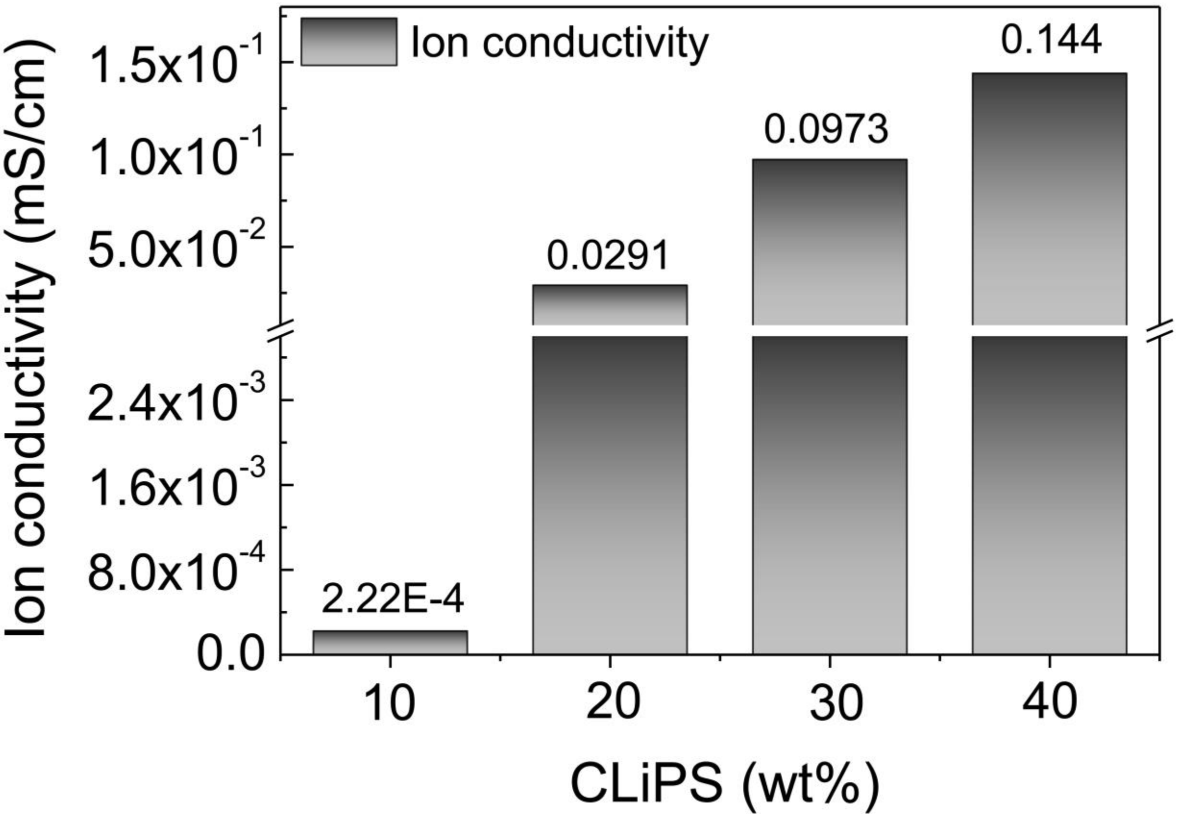

Supplementary Fig. 4 | Ion conductivity as a function of IL concentration. Ion conductivities of various CLiPS (10-40 wt%) calculated from electrochemical impedance spectroscopy (EIS) data under maximum pressure conditions.

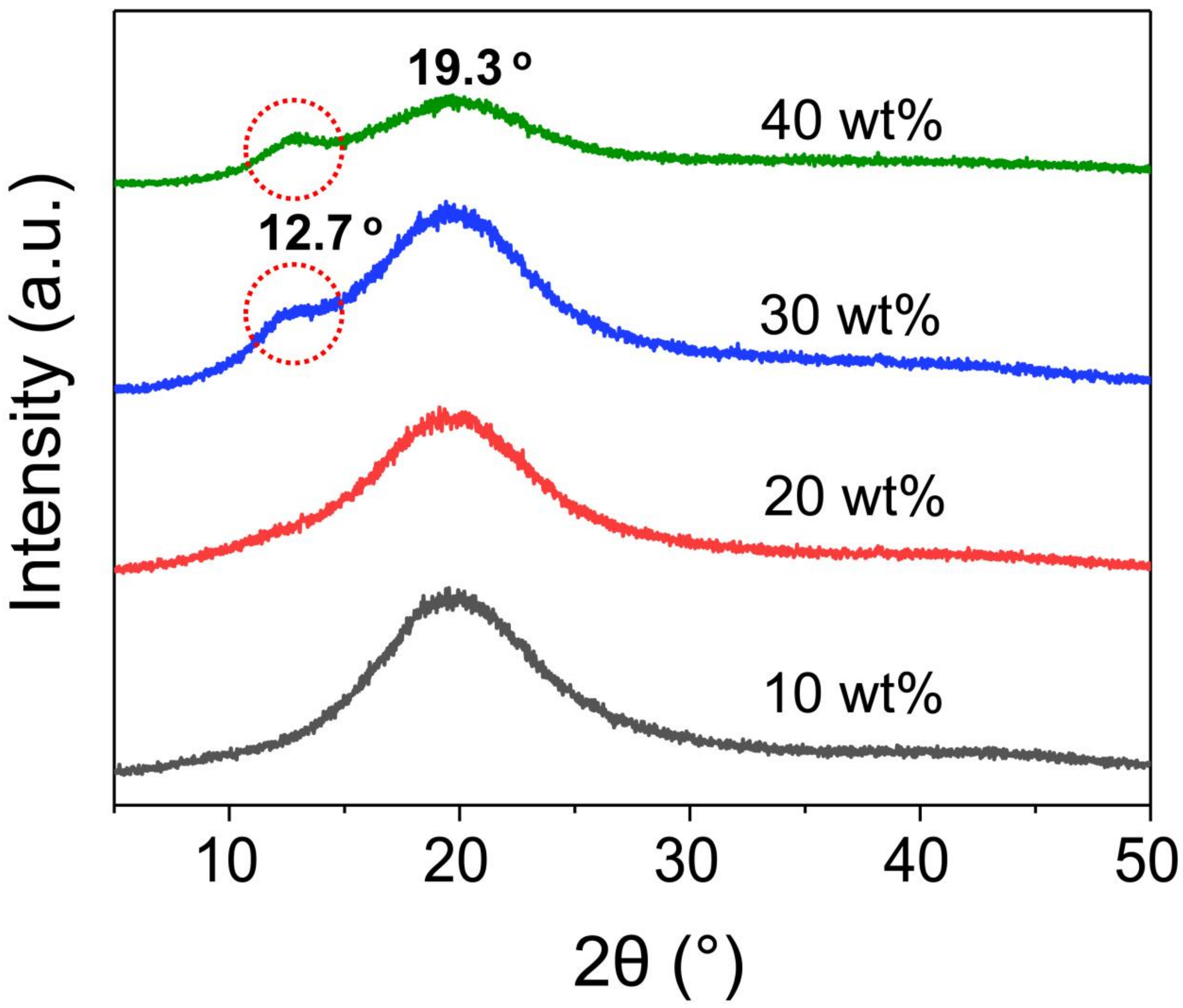

Supplementary Fig. 5 | XRD graph patterns of CLiPS with varying IL concentration (10-40 wt%) indicating intercalation of ions in the hard segment (prominent in 30 and 40 wt%).

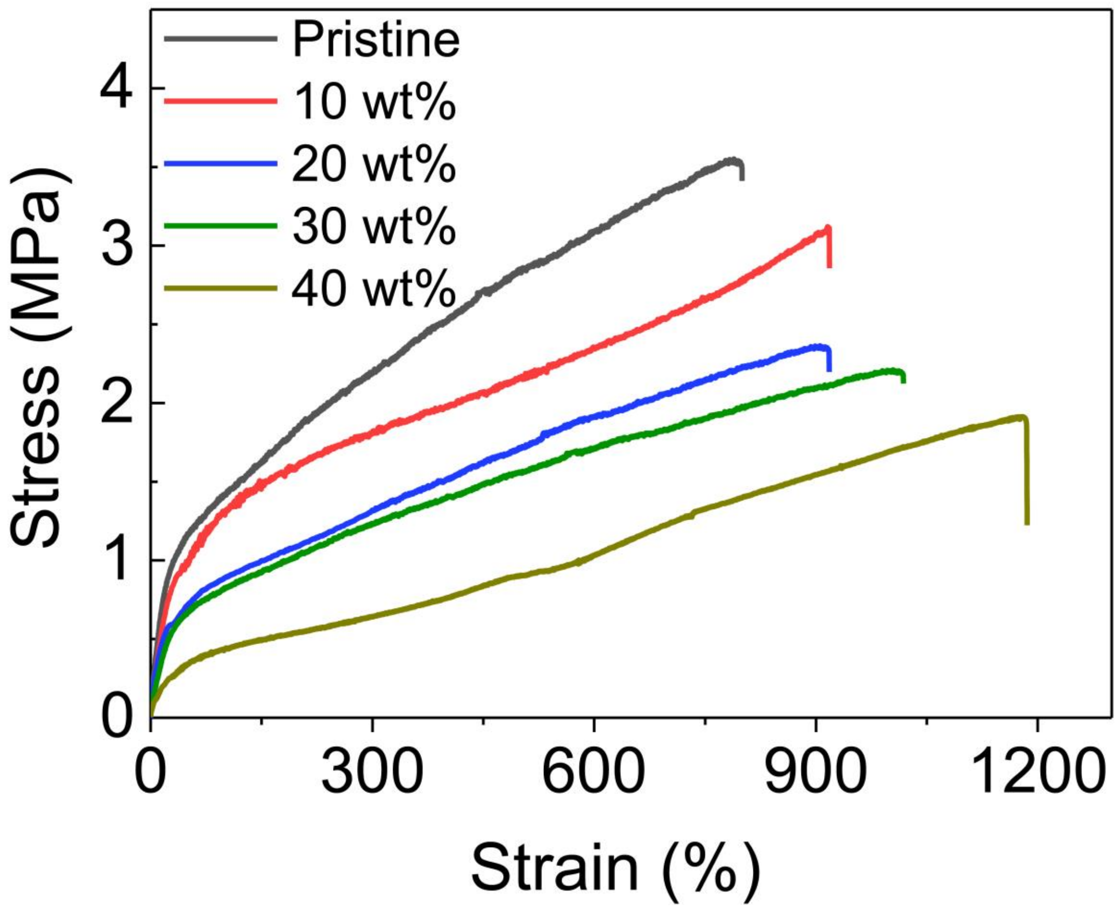

Supplementary Fig. 6 | Stress-strain curves of CLiPS with varying IL concentration (0-40 wt%). Retention of excellent mechanical properties even as IL concentration increases.

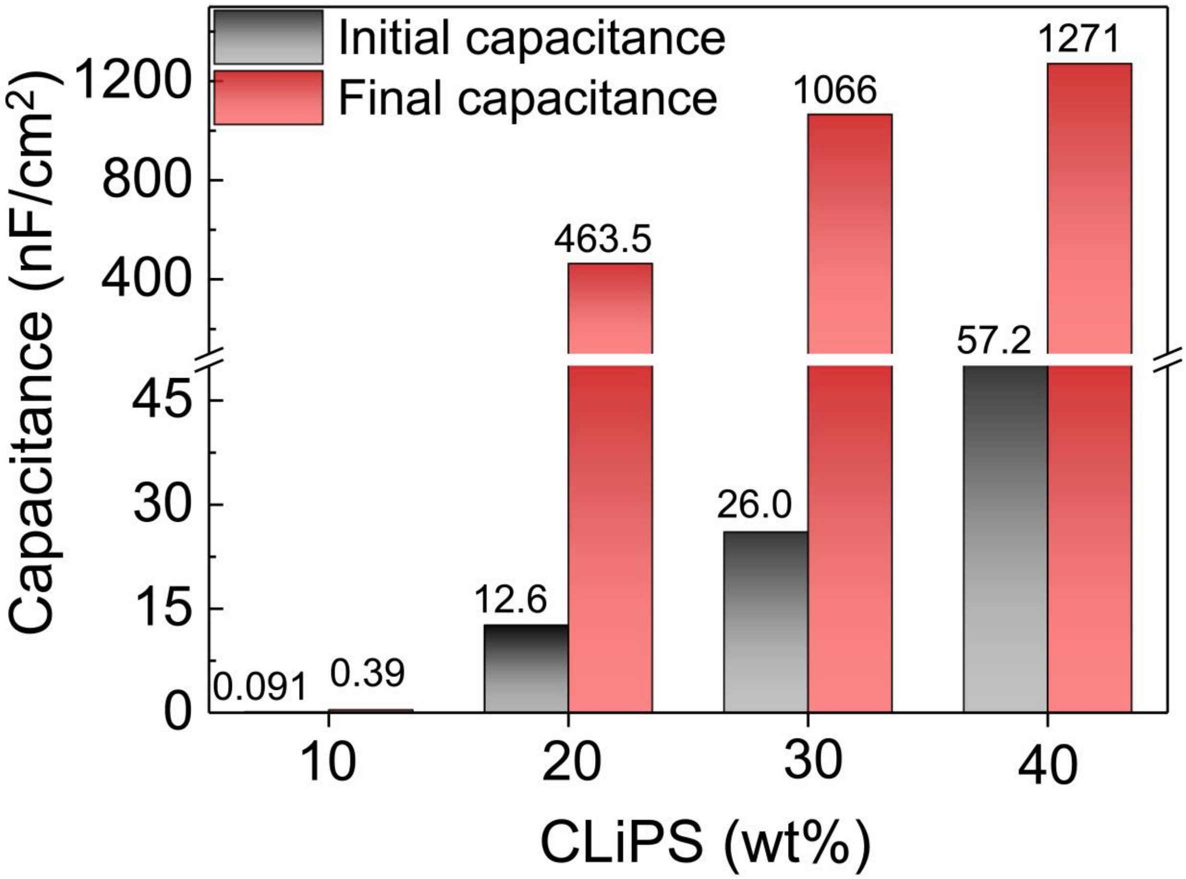

Supplementary Fig. 7 | Pressure-dependent capacitance changes of CLiPS. CLiPS with varying IL concentration (10-40 wt%) presenting the initial and final capacitance values, under pressure range of ~23 kPa (applied bias voltage of 1 V at 20 Hz).

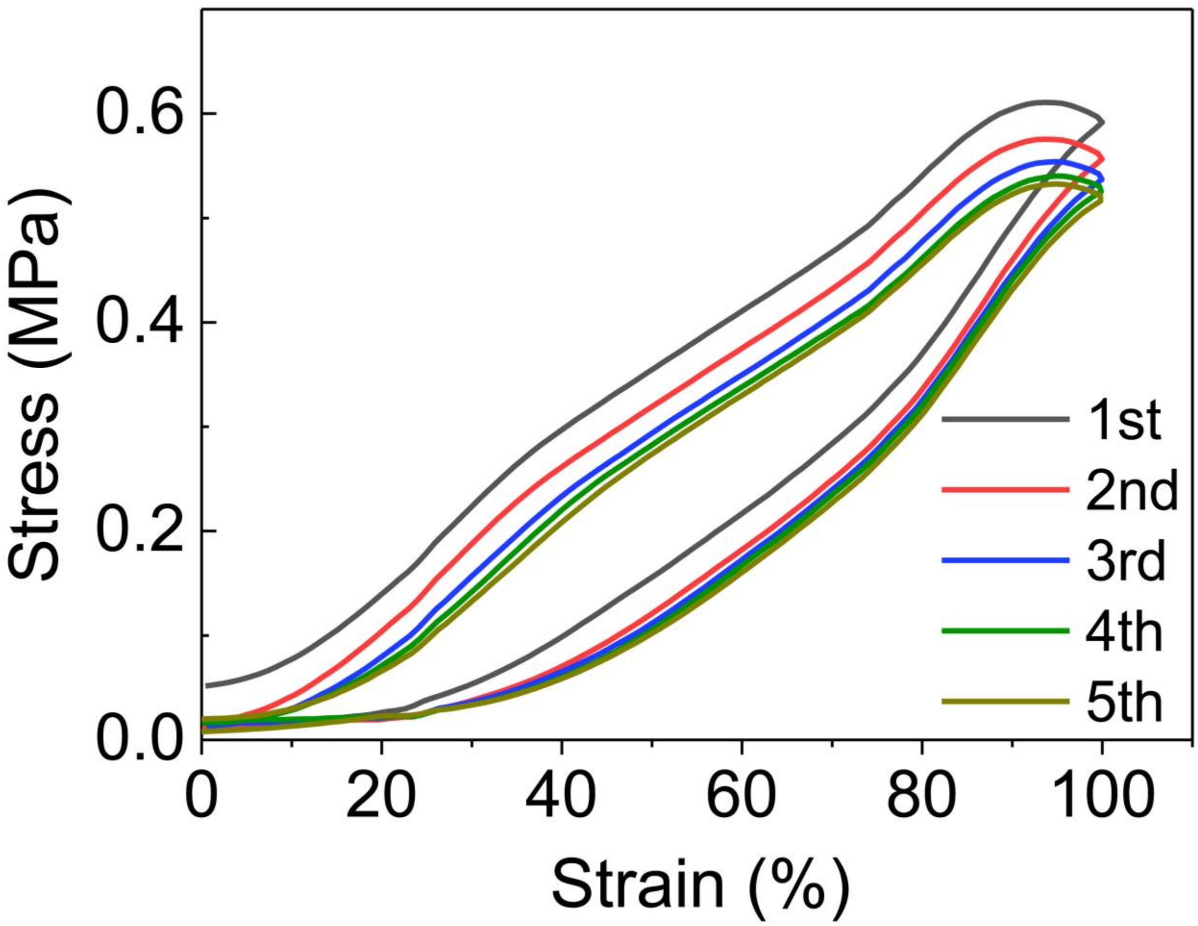

Supplementary Fig. 8 | Loading/unloading cycles at different strains. Stress-strain curves representing the excellent elastic recovery (100%) of CLiPS with five times loading and unloading cycles.

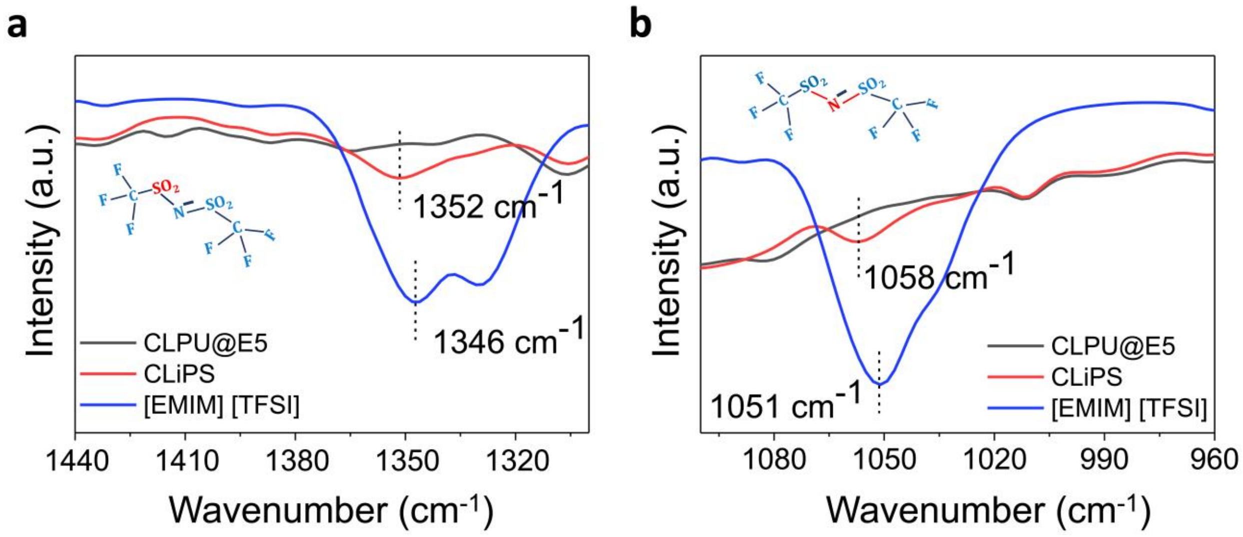

Supplementary Fig. 9 | ATR-FTIR characterization of $[EMIM]^+[TFSI]^-$, CLiPS and CLPU@E5 to verify ion-dipole interaction. **a, b,** range of 1440-960 cm^{-1} corresponding to $TFSI^-$ stretching in CLiPS due to the weakened Coulomb forces caused by ion-dipole interactions effect on the cations.

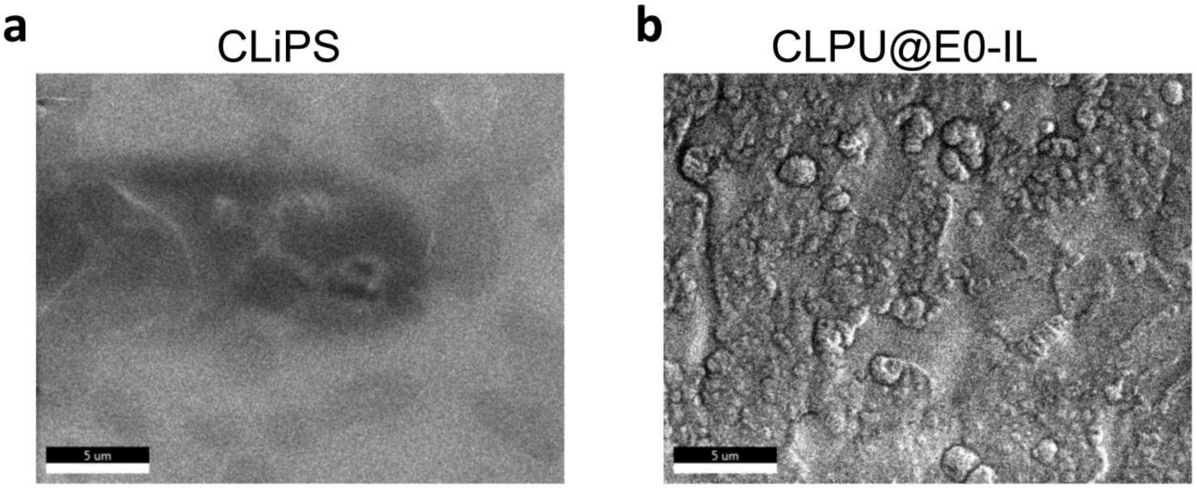

Supplementary Fig. 10 | Field emission scanning electron microscopy (FE-SEM) images. **a,** CLiPS showing uniform dispersion of ion pairs and **b,** CLPU@E0-IL showing ion aggregations (scar bar at 5 μm).

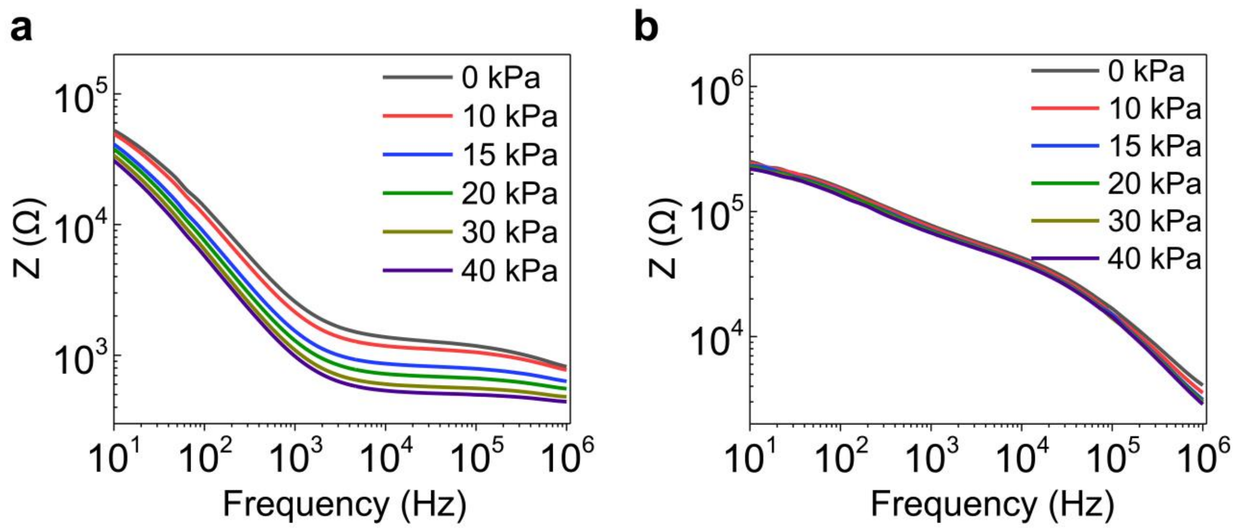

Supplementary Fig. 11 | Ion trap and release effect. Stress relaxation characterization in relation to bulk resistance behavior as a function of applied external pressure. **a,** CLiPS and **b,** CLPU@E0-IL.

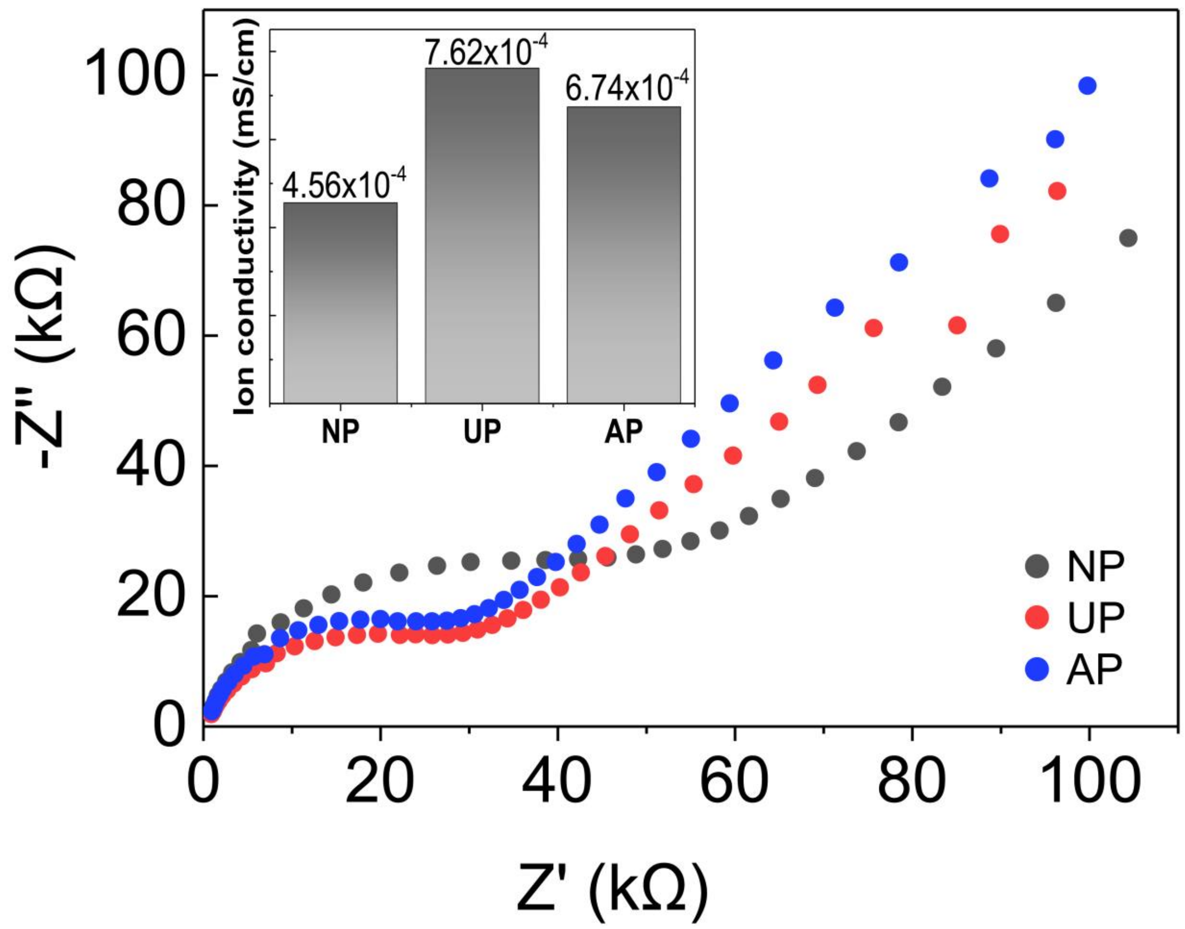

Supplementary Fig. 12 | Impedance Nyquist plots of CLPU@E0-IL. Nyquist plot of CLPU@E0-IL under no pressure (NP), under pressure (UP), and after removing pressure (AP), depicting no significant reversible movement of ions (insert shows ion conductivity under each condition).

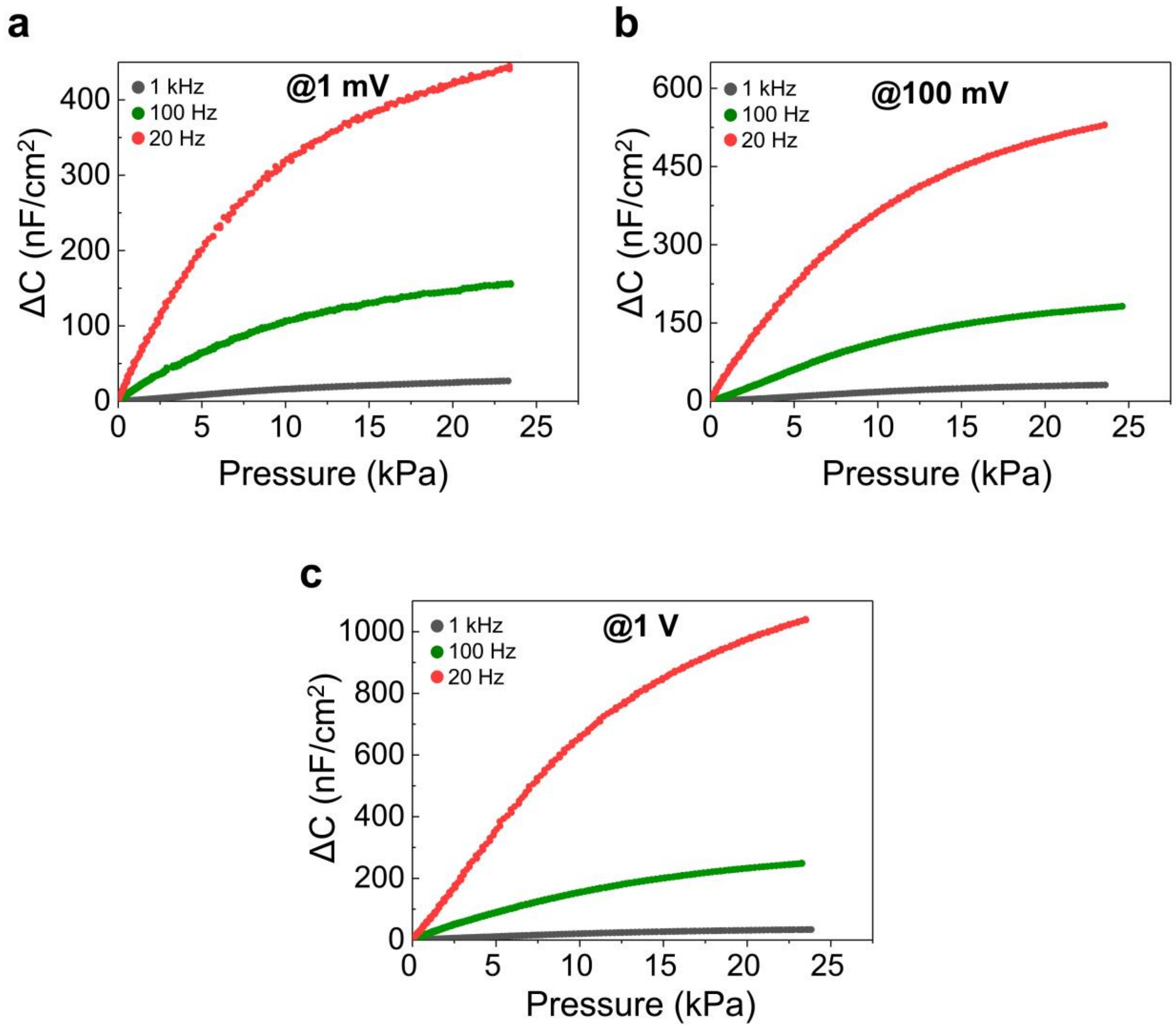

Supplementary Fig. 13 | Pressure response of CLiPS-based piezocapacitive sensors. Pressure-dependent capacitance changes of the CLiPS (30 wt%)-based device under different applied bias voltages (1 mV, 100 mV and 1 V) as a function of different frequencies (1 kHz, 100 Hz, and 20 Hz).

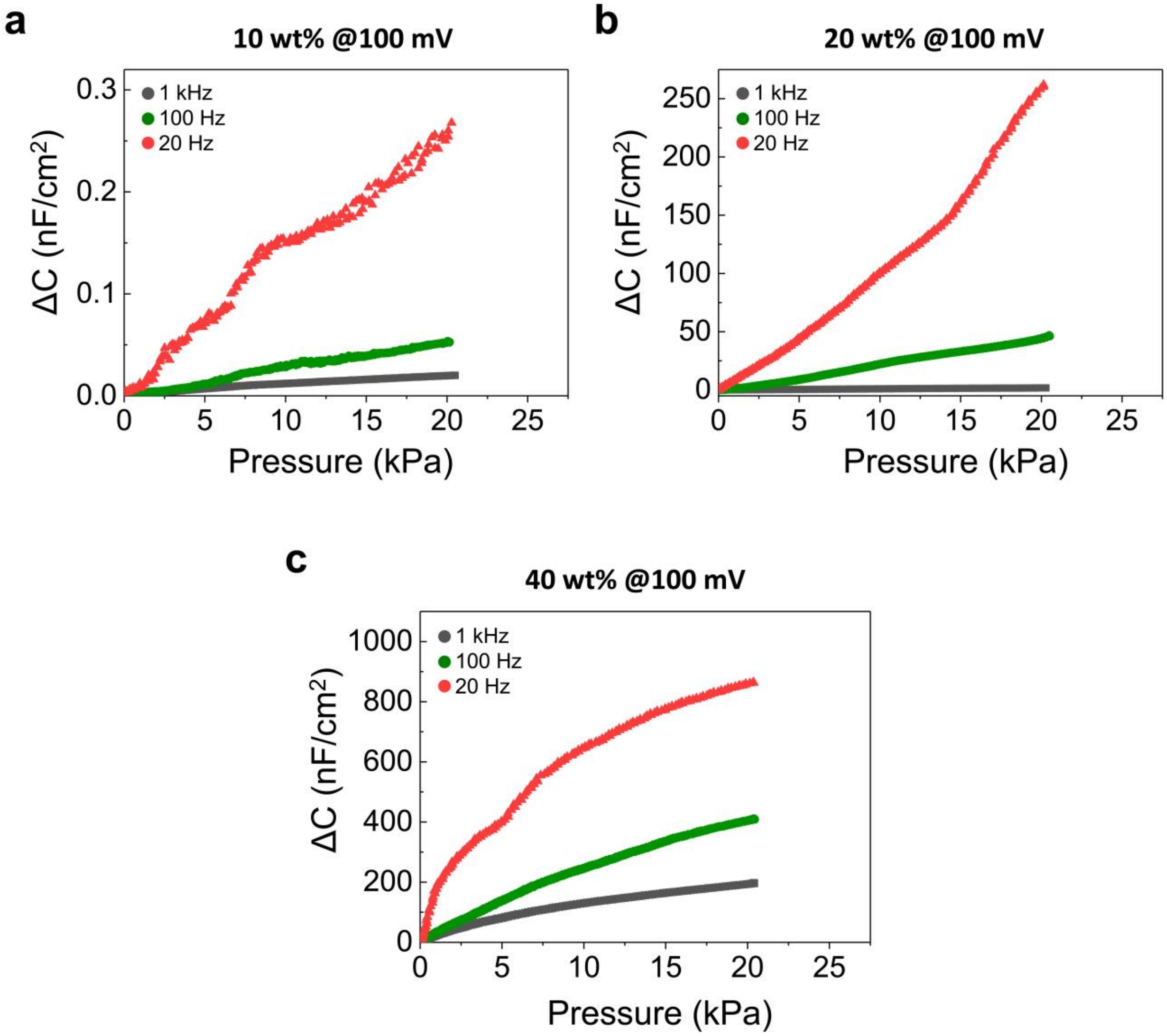

Supplementary Fig. 14 | Pressure response of CLiPS-based piezocapacitive sensors. Pressure-dependent capacitance changes of the CLiPS (10, 20, 40 wt%)-based device under applied bias voltage (100 mV) as a function of different frequencies (1 kHz, 100 Hz, and 20 Hz).

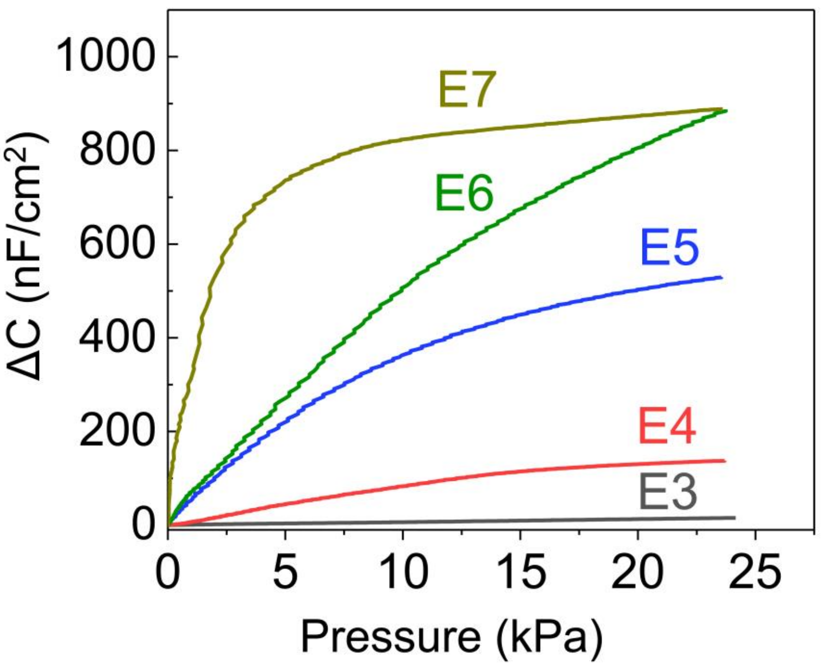

Supplementary Fig. 15 | Pressure response characteristics of CLPUs. Pressure-dependent capacitance changes of CLPUs (applied bias voltage 100 mV at 20 Hz) exhibiting a trend increase in capacitance changes with the increase of Cl groups content.

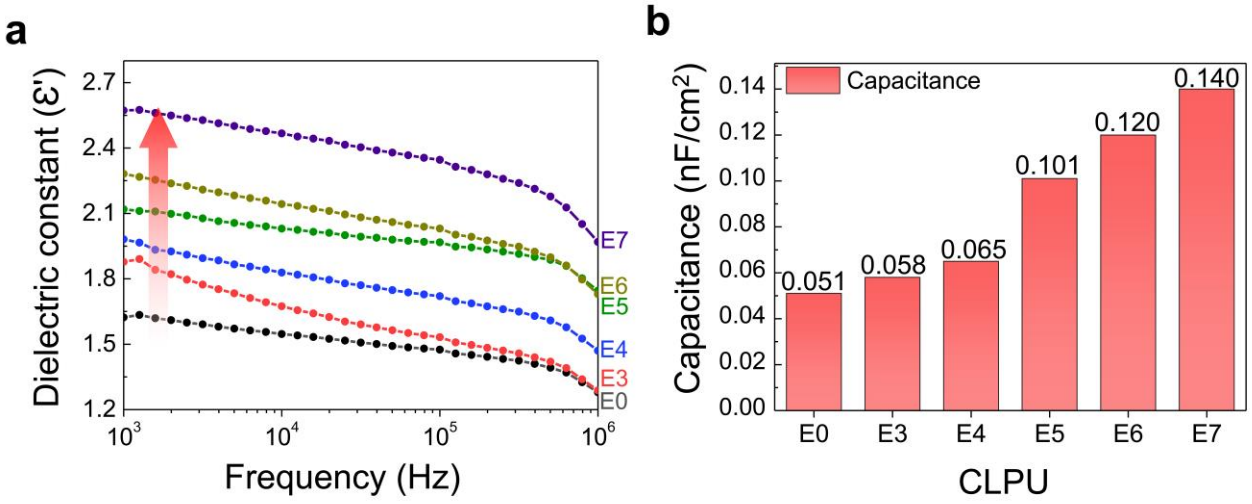

Supplementary Fig. 16 | Impart of Cl groups on the capacitance of CLiPS. a, Dielectric constants increases with the increase in Cl groups contents **b,** maximum capacitance values (applied bias voltage of 1 V at 1 kHz) of CLPU films without IL concentration, exhibiting an increasing trend with respect to Cl groups content.

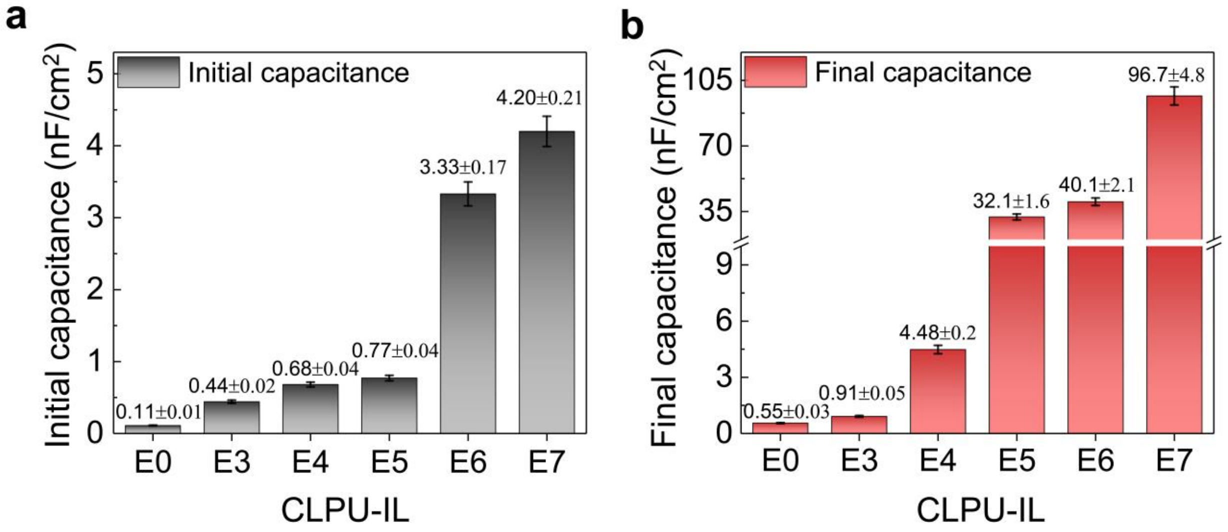

Supplementary Fig. 17 | Pressure-dependent behavior of CLPUs. Bar representation of **a,** initial capacitance (without pressure) and **b,** final capacitance under pressure ~23 kPa (applied bias voltage 100 mV at 1 kHz) as a function of Cl groups content (CLPU films with 30 wt% IL concentration).

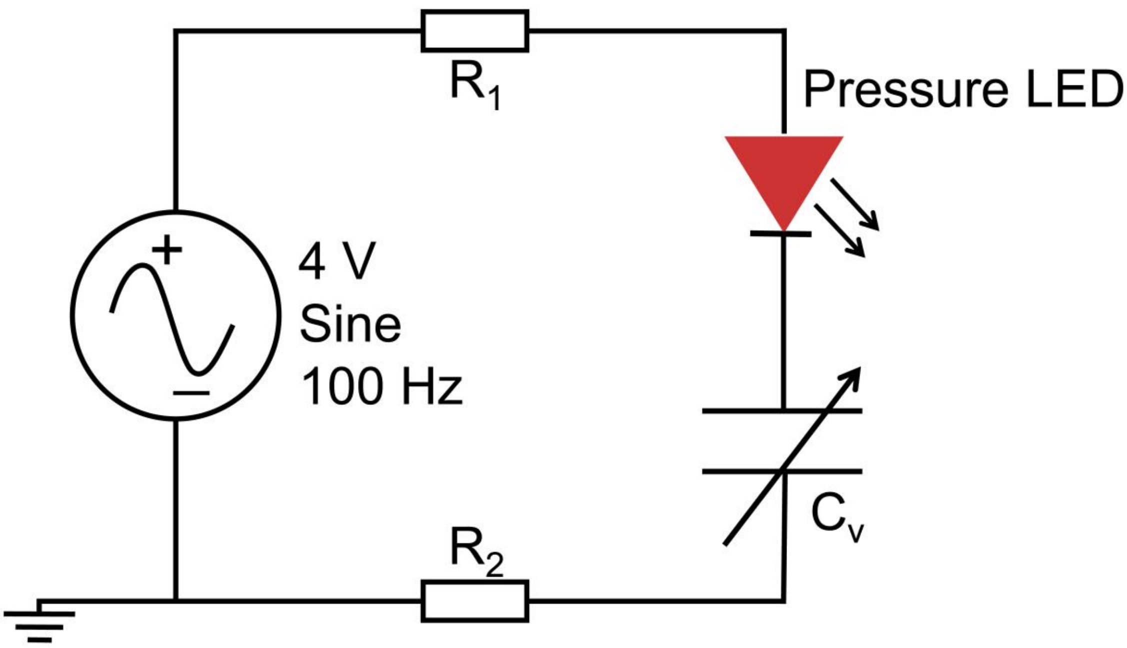

Supplementary Fig. 18 | Electrical circuit for pressure LED demonstration. Simple circuit of CLiPS- or CLPU@E0-IL-based device (C_v denotes variable capacitor, R_1 and R_2 denotes resistances of Ag wires) and LED connected in series.

Supplementary Table 1. Presentation of ECH and THF mole ratio which constitute the soft segment composition of the various synthesized CLPU and their molecular weights.

	**E0**	**E3**	**E4**	**E5**	**E6**	**E7**
**ECH**	0	36.9%	42.8%	50.8%	56.0%	72.0%
**THF**	100%	63.1%	57.2%	49.2%	44.0%	28.0%
**Mw**	2000	2605	2295	2115	2158	2026
**PDI**	1	1.25	1.19	1.16	1.14	1.27

Supplementary Table 2. Mechanical properties of CLPU films without IL (pristine)

Sample	Stress (MPa)	Strain (%)	Modulus (MPa)
E7-pristine	5.31	283	6.5
E6-pristine	4.35	503	5.3
E5-pristine	3.55	800	4.9
E4-pristine	1.82	757	2.4
E3-prisitine	1.28	958	0.5

Supplementary Table 3. Mechanical properties of CLPU films with 30 wt% IL concentration displaying decreased modulus and increased elongation at break

Sample	Stress (MPa)	Strain (%)	Modulus (MPa)
E7-30 wt% IL	3.68	302	4.2
E6-30 wt% IL	2.38	947	3.4
E5-30 wt% IL	2.20	1018	2.4
E4-30 wt% IL	0.95	1122	1.1
E3-30 wt% IL	0.81	1170	0.3

Supplementary Table 4. Mechanical properties and self-healing efficiencies of CLiPS films at various healing time intervals

Sample	Self-healing time (min)	Stress (MPa)	Elongation (%)	Modulus (MPa)	Healing efficiency (%)
CLiPS	Original	2.20	1018	2.4	-
CLiPS	20	0.60	249	0.4	27.3
CLiPS	40	1.24	610	1.8	56
CLiPS	60	2.0	994	2.1	91

Supplementary Table 5. Comparison of self-healing efficiency, autonomous capability, mechanical properties (elastic modulus and elongation at break), and sensitivity of previously reported self-healing iontronic pressure sensors.

Color band	Matrix	Self-healing efficiency (%)	Auton-omous	Elastic modulus (MPa)	Elongation at break (%)	Sensitivity (kPa^{-1})	Ref
Red	CLPU/[EMIM][TFSI]	91	Yes	2.4	1018	7.36	**This work**
Black	Siloxane oligomers/Fe^{2+}	88	Yes	1.53	58	0.33	40 [21]
Blue	TFEA/[EMIM][TFSI]	99	Yes	0.72	1820	0.01	32 [22]
Green	Hydroxypropyl cellulose/Al^{3+}	85	Yes	0.2	200	3.14	38 [23]
Dark-blue	Poly(acrylic acid)/Fe^{3+}	100	Yes	0.8	1500	4.5	52 [24]
Violet	OSA-CMC-AG0/Li^{+}	90	Yes	0.1	40	1.15	53 [25]
Turqu-oise	PU/[DEIM][TFSI]	95	No	0.42	327	0.37	33 [26]

Supplementary References

1. Choi, J. *et al.* Synthesis of highly functionalized thermoplastic polyurethanes and their potential applications. *Polymer (Guildf).* **116**, 287–294 (2017).
2. Yoneda, H. Effect of Substitution on Dipole Moments of Molecules. *Bull. Chem. Soc. Jpn.* **31**, 708–714 (1958).
3. Mcgivern, W. S., Derecskei-Kovacs, A., North, S. W. & Francisco, J. S. Computationally Efficient Methodology to Calculate C-H and C-X (X) F, Cl, and Br) Bond Dissociation Energies in Haloalkanes. (2000) doi:10.1021/jp993275d.
4. Blundell, D. J., Eeckhaut, G., Fuller, W., Mahendrasingam, A. & Martin, C. Real time SAXS/stress–strain studies of thermoplastic polyurethanes at large strains. *Polymer (Guildf).* **43**, 5197–5207 (2002).
5. Ying, W. Bin *et al.* A Biologically Muscle-Inspired Polyurethane with Super-Tough, Thermal Reparable and Self-Healing Capabilities for Stretchable Electronics. *Adv. Funct. Mater.* **31**, 2009869 (2021).
6. Chen, C. *et al.* A Self-Healing and Ionic Liquid Affiliative Polyurethane toward a Piezo 2 Protein Inspired Ionic Skin. *Advanced Functional Materials* (2021) doi:10.1002/adfm.202106341.
7. Yang, Y. & Urban, M. W. Self-healing polymeric materials. *Chem. Soc. Rev.* **42**, 7446–7467 (2013).
8. Jin, M. L. *et al.* An Ultrasensitive, Visco-Poroelastic Artificial Mechanotransducer Skin Inspired by Piezo2 Protein in Mammalian Merkel Cells. *Adv. Mater.* **29**, (2017).
9. Kim, S. M. *et al.* Superior Toughness and Fast Self-Healing at Room Temperature Engineered by Transparent Elastomers. *Adv. Mater.* **30**, 1–8 (2018).
10. Xu, W. *et al.* A Multiscale Investigation on the Mechanism of Shape Recovery for IPDI to PPDI Hard Segment Substitution in Polyurethane. *Macromolecules* **49**, 5931–5944 (2016).
11. Cao, Y. *et al.* A Transparent, Self-Healing, Highly Stretchable Ionic Conductor. *Adv. Mater.* **29**, 1–9 (2017).
12. Zhang, Y. *et al.* Highly Transparent, Underwater Self-Healing, and Ionic Conductive Elastomer Based on Multivalent Ion-Dipole Interactions. *Chem. Mater.* **32**, 6310–6317 (2020).
13. Pal, P. & Ghosh, A. Ion conduction and relaxation mechanism in ionogels embedded with imidazolium based ionic liquids. *J. Appl. Phys.* **126**, (2019).
14. Singh, M. B. & Kant, R. Debye-Falkenhagen dynamics of electric double layer in presence of electrode heterogeneities. *Journal of Electroanalytical Chemistry* vol. 704 197–207 (2013).
15. Kortschot, R. J., Philipse, A. P. & Erné*erné*, B. H. Debye Length Dependence of the Anomalous Dynamics of Ionic Double Layers in a Parallel Plate Capacitor. (2014) doi:10.1021/jp5025476.
16. Singh, M. B. & Kant, R. *Theory of Electric Double Layer Dynamics at Blocking Electrode*. (2011).
17. You, I. *et al.* Artificial multimodal receptors based on ion relaxation dynamics. *Science (80-.).* **370**, 961–965 (2020).
18. Joshi, J. H., Kanchan, D. K., Joshi, M. J., Jethva, H. O. & Parikh, K. D. Dielectric

relaxation, complex impedance and modulus spectroscopic studies of mix phase rod like cobalt sulfide nanoparticles. *Mater. Res. Bull.* **93**, 63–73 (2017).
19. Liang, J. *et al.* Structure and dynamics of ions in dipolar solvents: a coarse-grained simulation study †. *Cite this Soft Matter* **17**, 6305 (2021).
20. Wang, T. *et al.* Electroactive polymers for sensing. *Interface Focus* **6**, (2016).
21. Pignanelli, J. *et al.* Imine and metal-ligand dynamic bonds in soft polymers for autonomous self-healing capacitive-based pressure sensors †. (2019) doi:10.1039/c9sm01254k.
22. Xu, L. *et al.* A Transparent, Highly Stretchable, Solvent-Resistant, Recyclable Multifunctional Ionogel with Underwater Self-Healing and Adhesion for Reliable Strain Sensors. (2021) doi:10.1002/adma.202105306.
23. Bai, L. *et al.* Highly synergistic, electromechanical and mechanochromic dual-sensing ionic skin with multiple monitoring, antibacterial, self-healing, and anti-freezing functions †. (2021) doi:10.1039/d1ta06798b.
24. Ali Darabi, M. *et al.* Skin-Inspired Multifunctional Autonomic-Intrinsic Conductive Self-Healing Hydrogels with Pressure Sensitivity, Stretchability, and 3D Printability. *Adv. Mater* **29**, 1700533 (2017).
25. Wang, Y., Chen, Z., Chen, R. & Wei, J. A self-healing and conductive ionic hydrogel based on polysaccharides for flexible sensors. *Chinese J. Chem. Eng.* (2022) doi:10.1016/j.cjche.2022.02.022.
26. Li, T. *et al.* Mechanically Robust, Elastic, and Healable Ionogels for Highly Sensitive Ultra-Durable Ionic Skins. (2020) doi:10.1002/adma.202002706.
