# Supplementary material for: Ultrafast, autonomous self-healable iontronic skin exhibiting piezo-ionic dynamics
Source: Nat Commun. 2022 Dec 13;13:7699. doi: 10.1038/s41467-022-35434-8 (PMC9744819; doi:10.1038/s41467-022-35434-8)
Supplement: Supplementary file 2 — Description of Additional Supplementary Files [file 41467_2022_35434_MOESM2_ESM.pdf]

Description of Additional Supplementary Files

File Name: Supplementary Movie 1
Description: **Scratch test on CLiPS.** This movie presents the cross-section view of the fast autonomous self-healing capability of CLiPS.

File Name: Supplementary Movie 2
Description: **Tractive self-healing of electrode.** This movie presents the self-healing of the electrode demonstrated by connecting to an LED.

File Name: Supplementary Movie 3
Description: **Modulation of LED brightness with CLiPS.** This movie presents the demonstration of CLiPS-based device as a pressure-induced tactile sensor to modulate the brightness of LED.

File Name: Supplementary Movie 4
Description: **No change in LED brightness with CLPU@E0-IL.** This movie presents the demonstration of CLPU@E0-IL-based device exhibiting no significant change in LED intensity under pressure.”
